# *Pseudomonas* virulence factor SaxA detoxifies plant glucosinolate hydrolysis products, rescuing a commensal that suppresses virulence gene expression

**DOI:** 10.1093/ismeco/ycag004

**Published:** 2026-01-09

**Authors:** Kerstin Unger, Rebecca Ruiter, Michael Reichelt, Jonathan Gershenzon, Matthew T Agler

**Affiliations:** Institute of Microbiology, Plant Microbiosis Lab, Friedrich Schiller University Jena, 07743 Jena, Germany; Institute of Microbiology, Plant Microbiosis Lab, Friedrich Schiller University Jena, 07743 Jena, Germany; Department of Biochemistry, Max-Planck-Institute for Chemical Ecology, 07743 Jena, Germany; Department of Biochemistry, Max-Planck-Institute for Chemical Ecology, 07743 Jena, Germany; Institute of Microbiology, Plant Microbiosis Lab, Friedrich Schiller University Jena, 07743 Jena, Germany; Cluster of Excellence Balance of the Microverse, Friedrich Schiller University Jena, 07743 Jena, Germany

**Keywords:** SaxA, sulforaphane, ITC hydrolase, public good, glucosinolates, isothiocyanates, detoxification, phyllosphere, plant pathogen

## Abstract

Plants produce a plethora of specialized metabolites that often play important roles in their defence against pathogenic microbes or herbivorous insects. Exposure of leaf-colonizing microbes to these metabolites influences their growth, and we hypothesize that it also has consequences for microbe–microbe interactions. In Brassicaceae plants like the model plant *Arabidopsis thaliana*, glucosinolates and their biologically active derivatives, the isothiocyanates, are major defence metabolites. Adapted plant pathogens like *Pseudomonas* spp. use the hydrolase SaxA to convert the antimicrobial isothiocyanate sulforaphane to a non-toxic amine, whereas non-adapted commensal microbes are inhibited by this plant toxin. We used *Plantibacter* sp. 2H11-2 as a model commensal in co-culture with either *Pseudomonas viridiflava* 3D9 wildtype or a *saxA*-knock-out mutant. Both strains were isolated from the same wild *A. thaliana* population. Without isothiocyanate, *Plantibacter* grew better alone than with *Pseudomonas*, a potential competitor. At high isothiocyanate concentrations, however, the commensal was dependent on SaxA-mediated isothiocyanate degradation in both solid and liquid medium. At intermediate isothiocyanate concentrations, *Plantibacter*’s transcriptome changed in response to sulforaphane in monoculture but not in co-culture with *Pseudomonas*, suggesting that it was fully protected from this toxin. In return, *Plantibacter* caused transcriptional changes in *Pseudomonas*, suppressing biofilm formation and increasing amino acid metabolism gene expression which might suppress virulence and so contribute to plant health. Together, we find that degradation of an antimicrobial plant metabolite can protect a commensal to depend on a pathogen-produced virulence factor, suggesting effects on community composition in environments where microbes are exposed to ITCs.

## Introduction

Plants produce and secrete a variety of specialized metabolites that are probably best known for their antimicrobial effects and defence against pathogenic microbes [1–4]. For example, plant specialized metabolites influence microbial colonization and infection of leaves [5, 6]. Outside of plants, they also influence the soil quality [7, 8] and feeding behaviours of herbivorous insects [9]. As a reaction to plant defence molecules, some microbes evolved enzymes to detoxify these metabolites [6, 10–12] which may change the chemical landscape of an environment for example of a leaf [13] or of soils [14] or insect guts [15, 16] where plant leaves are decayed or digested. We hypothesize that plant specialized metabolites could have a large influence on microbial interactions and hence microbiome structures in several environments, but few investigations have covered this topic. Microbial interactions can also be mediated by better known mechanisms, such as competition for nutrients [17], production of antimicrobial substances to inhibit competitors [18], and metabolite cross-feeding [19]. To specifically study the effect of plant specialized metabolite detoxification on microbial interactions, we chose a glucosinolate-derived defence metabolite which occurs in *Arabidopsis thaliana* as a model.

Glucosinolates (GLS) are defence metabolites in Brassicaceae plants like *A. thaliana* as well as various crops, including *Brassica oleracea*, where they are stored in large amounts in leaves [20, 21]. GLS themselves have no antimicrobial effect, but they are activated by hydrolytic breakdown catalysed by myrosinases (β-glucosidases) [22]. The most studied GLS breakdown products are isothiocyanates (ITCs), which have antimicrobial activity against diverse pathogenic leaf colonizers [2, 6, 11, 23], deter herbivorous insects [24, 25], and restructure microbial communities in soil [8, 14]. Microbes in these environments are thought to be exposed to a range of different ITC concentrations. For example, in *A. thaliana* leaves, on the one hand, high levels of ITCs are locally released when pathogens cause necrotic lesions or herbivorous insects bite into the leaf and myrosinases come in contact with GLS [22, 26]. On the other hand, low levels of ITCs are suggested to be constantly present inside the leaf apoplast because of constant turnover of GLS as part of plant sulphur cycling [27]. Exposure of leaf bacteria to ITCs may also happen on the leaf surface when bacteria metabolize GLS [28]. Here, we focus on 4MSOB-ITC (4-methylsulfinylbutyl-ITC, sulforaphane) which is the major breakdown product of GLS in the widely used reference *A. thaliana* genotype Col-0. It was shown to reduce loads of non-adapted *Pseudomonas* spp., *Xanthomonas* spp., or *Pectobacterium* spp. in leaves and inhibit bacterial growth *in vitro* at high levels (up to 200 μg/ml) [11, 29]. Additionally, very low levels of 4MSOB-ITC (20 μM = 3.5 μg/ml) already reduce the virulence of plant pathogens like *Pseudomonas syringae* and *Xanthomonas campestris* [30, 31].

ITCs represent a major barrier to microbial colonization, so that is presumably why some well-adapted *A. thaliana* pathogens express *sax* genes (survival in *Arabidopsis* extract). These genes encode efflux pumps (*saxF*, *saxG*, *saxD*), an uncharacterized protein (*saxB*), a transcriptional regulator (*saxC*), and an ITC hydrolase (*saxA*) [11, 32]. The SaxA protein degrades a broad spectrum of ITCs including 4MSOB-ITC to their corresponding amines [32] rendering them non-toxic [6, 11]. In various pathosystems, SaxA functions as a virulence factor increasing pathogen growth and disease severity [11, 29–31], but the ecological role of SaxA during pathogenesis and in other environments is unknown. We hypothesize that it is important: Although the effects of ITCs on microbes beyond plant pathogens are less studied, they are broadly toxic due to unspecific modes of action like disturbing protein structures, impairing membrane stability, or general stress responses [33–35]. In a recent study, we demonstrated growth inhibition of several commensal leaf bacterial isolates, including *Plantibacter* spp., by 4MSOB-ITC and allyl-ITC *in vitro* [28].

**Figure 1:**
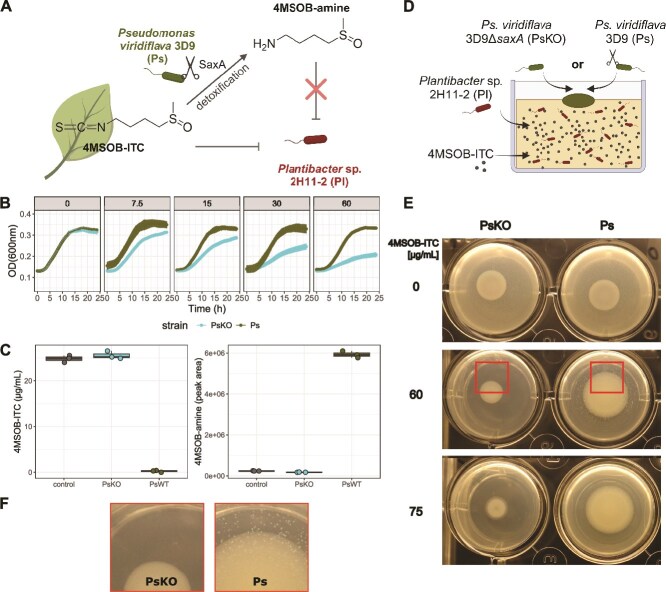
SaxA as public good in the interaction between *Pseudomonas viridiflava* 3D9 (Ps) and *Plantibacter* sp. 2H11-2 (Pl). (**A**) Schematic drawing of the research hypothesis. *Arabidopsis thaliana* Col-0 produces 4MSOB-ITC as defence compound, plant pathogen *P. viridiflava* 3D9 (Ps) can degrade it to 4MSOB-amine. The growth of commensal *Planibacter* sp. 2H11-2 (Pl) is inhibited by 4MSOB-ITC but not by 4MSOB-amine. Thus, we hypothesize that SaxA-mediated ITC degradation functions as public good which benefits not only Ps but additionally Pl. Created in BioRender. Unger, K. (2026) https://BioRender.com/7h0un29. (**B**) Growth curves of Ps and PsKO (Ps 3D9Δ*saxA*) with 4MSOB-ITC concentrations from 0 to 60 μg/ml (mean ± standard deviation of *n* = 3 are shown). (**C**) Quantification of 4MSOB-ITC and its breakdown product 4MSOB-amine by LC–MS in supernatants of bacterial overnight cultures (*n* = 3). Non-inoculated R2A broth supplemented with 30 μg/ml 4MSOB-ITC served as control. (**D**) Public good assay: schematic cross-section of the agar plate assay to visualize a public good effect of Ps on Pl in 4MSOB-ITC background. 4MSOB-ITC at different concentrations and Pl cells in low density (OD = 0.001) were mixed in R2A agar and poured in wells of a 24-well plate. Then, 2-μl drop spots of Ps or PsKO were placed on top of the agar layer and growth was observed after 4 days of incubation at 30°C. Created in BioRender. Unger, K. (2026) https://BioRender.com/f0cc08r. (**E**) growth of Pl (in the agar) and Ps and PsKO (colony in the middle) at different 4MSOB-ITC concentrations. (**F**) Close-up of PsKO/Pl and Ps/Pl (boxes marked in E).

Understanding the interaction dynamics between leaf pathogens and commensals is important because commensal bacteria can prevent plant disease by pathogen suppression [36, 37], which might help to improve plant health and also increase the effectiveness of biocontrol agents [38, 39]. The reverse effect of pathogens on commensals is less well known but likely as important for understanding the consequences of these interactions. Since pathogens and commensals can be isolated together from healthy *A. thaliana* leaves [40], we hypothesize that plant specialized metabolites like ITCs play a major role in pathogen–commensal interactions. Specifically, we hypothesized that SaxA-mediated ITC degradation could serve as a public good that not only benefits the pathogenic degrader strain but also non-degrading commensal bacteria. We investigate this hypothesis (Fig. 1A) and its temporal and spatial implications using the opportunistic pathogen *Pseudomonas viridiflava* 3D9 (hereafter: Ps), which degrades ITCs via SaxA [28] and was co-isolated from wild *A. thaliana* plants together with the ITC-sensitive, commensal strain *Plantibacter* sp. 2H11-2 (hereafter: Pl) [40].

## Material and methods

### Strains and culture conditions

*Pseudomonas viridiflava* 3D9 (Ps) and *Plantibacter* 2H11-2 (Pl) were isolated from wild *A. thaliana* leaves in 2019 [28, 40]. In whole genomes, we found homologues of *sax* genes in Ps, but not in Pl. Namely, Ps contains one copy each of the ITC-hydrolase gene *saxA*, the transcriptional regulator gene *saxC*, *saxB* with unknown function, and the efflux systems *saxF* and *saxG*. Additionally, we annotated two copies of the efflux system *saxD* [28]. *Pseudomonas viridiflava* 3D9Δ*saxA* (hereafter: PsKO) was generated as part of this study. If not mentioned otherwise, all strains were grown in Reasoner's 2A (R2A) broth or on R2A agar (yeast extract 0.5 g/l, peptone 0.5 g/l, casein hydrolysate 0.5 g/l, glucose 0.5 g/l, soluble starch 0.5 g/l, K_2_HPO_4_ 0.3 g/l, MgSO_4_ 0.024 g/l, sodium pyruvate 0.3 g/l, if applicable: 1.5 g/l agar; pH = 7.2 ± 0.2 [41]) at 30°C. Throughout the experiments, we used 4MSOB-ITC (L-sulforaphane, CAS 142825-10-3, ≥95%; Sigma Aldrich) dissolved in DMSO (dimethyl sulfoxide; Carl Roth).

### Knock-out of *saxA* in *P. viridiflava* 3D9

To knock out *saxA* in Ps, we used a heterologous recombination protocol [42]. Details are described in the Supplementary Methods.

### Agar-based public good assay

R2A agar was cooled down to 45°C and Pl overnight culture was normalized to OD_600_ = 0.01 before mixing it into the warm agar in a final concentration of OD_600_ = 0.001. 4MSOB-ITC or DMSO was added in concentrations ranging from 0 to 75 μg/ml, and 1 ml of medium with Pl and ITC was poured into individual wells of a 24-well plate. Ps and PsKO pre-cultures were normalized to OD_600_ = 0.2 and 2 μl was drop spotted in the middle of a well as soon as the agar had solidified. The plate was incubated at 30°C for up to 4 days. The experiment was independently repeated twice.

### Bacterial co-culture experiments

#### Growth curves and endpoint bacterial loads

Overnight cultures of Ps, Pl, and PsKO were normalized to OD_600_ = 0.4, and equal volumes of two strains or one strain and pure R2A broth were mixed to generate the inocula. Then, 90 μl R2A broth with 4MSOB-ITC in concentrations ranging from 0 to 60 μg/ml was pipetted in individual wells of a 96-well plate. Ten microlitres of normalized bacterial cultures was used as inoculum. Thus, each strain was present at OD_600_ = 0.02 in each well. R2A broth served as non-inoculated control. The plate was sealed with transparent foil to prevent evaporation of the ITC, and the OD_600_ was measured every 15 min after 1 min of orbital shaking and recorded using the software i-control 2.0. The raw data were processed with custom scripts in R. After 22 h of incubation, a 10-fold serial dilution was prepared from each sample and 20 μl of up to three dilution steps per sample was spread on R2A plates. For 0–30 μg/ml, it was sufficient to plate the co-cultures on simple R2A plates. Ps and Pl can be differentiated based on the timepoint of appearance of the colony morphology (Supplementary Fig. S1). For 60 μg/ml, however, the count of Pl was much less than the Ps or PsKO count, and therefore we plated the co-cultures on selective R2A plates supplemented with 1.5 μg/ml kanamycin where Pl grew but Ps or PsKO were inhibited. Pl counts on R2A + Kan were normalized to counts on R2A to make all conditions comparable. The experiment was repeated once with three technical replicates.

#### Bacterial loads and 4MSOB-ITC and 4MSOB-amine analysis over time

As described for the growth curves, bacterial overnight cultures were normalized to OD_600_ = 0.4 and mixed in equal amounts or with R2A broth. The cultures were started with 0, 30, or 60 μg/ml 4MSOB-ITC in a total volume of 500 μl. They were incubated at 30°C at 200 rpm and sampled in regular intervals. At each sampling timepoint, 10 μl was taken and a 10-fold serial dilution was prepared in phosphate-buffered saline (PBS) and 20 μl per dilution step and samples were plated on R2A agar. Additionally, 20 μl of the supernatants was mixed with 180 μl Milli-Q water and frozen immediately at −20°C for 4MSOB-ITC and 4MSOB-amine quantification. The experiment shown in the main figure was independently repeated twice with three technical replicates.

#### Endpoint bacterial loads in leaf extract medium

To mimic the metabolic landscape of wounded leaves, e.g. caused by herbivores or pathogens, when GLS breakdown products are naturally released, we produced leaf extract medium according to Fan *et al*. [11]. The medium was produced either from *A. thaliana* plants with natural GLS content (Col-0, mainly 4MSOB-GLS) or without aliphatic GLS (mutant plant *myb28/29* [43]). Leaves of 4-week-old plants were crushed in an equal volume of R2A broth (1 mg leaf = 1 μl R2A broth) to release GLS breakdown product. The resulting leaf extract medium was centrifuged for 10 min at 7500×*g* and filter sterilized (0.2 μm filter) to remove plant debris and microbes. The medium was frozen at −20°C and filtered again before use. Overnight cultures of Ps, Pl, and PsKO were normalized to OD_600_ = 0.4, and equal volumes of two strains or one strain and pure R2A broth were mixed to generate the monoculture and co-culture inocula. Then, 90 μl leaf extract medium supplemented with either 60 μg/ml 4MSOB-ITC (in *myb28/29* extracts) or pure DMSO as controls (in *myb28/29* and Col-0 extracts) was pipetted in individual wells of a 96-well plate (*n* = 3 technical replicates). Ten microlitres of normalized bacterial cultures was used as inoculum to achieve a starting OD_600_ = 0.02 for each strain in each well. R2A broth served as non-inoculated control. The plate was sealed with transparent foil to prevent evaporation of the ITC and incubated at 30°C on the shaker at 220 rpm for 23–24 h. To determine bacterial counts, a 10-fold dilution series was prepared from each well and 5 μl was drop spotted on R2A (Ps, PsKO counts) or R2A + 1.5 μg/ml kanamycin (Pl counts) in duplicates. Ps and PsKO were counted after 24 h at 30°C, and Pl was counted after 48 h at 30°C (Supplementary Fig. S1). The experiment was repeated three times with three technical replicates, each time with leaf extract medium produced from independently grown batches of plants.

#### Bacterial loads *in planta*

To test the effects of Ps or *saxA* on Pl growth in living leaf tissues, we used gnotobiotic *A. thaliana* Col-0 and *myb28/29* plants in a flow pot system similar to that of Kremer *et al*. [44], inoculated them with either Pl monocultures or co-cultures Ps + Pl or PsKO + Pl, and counted the CFUs 24 h after infection. The details are described in the Supplementary Methods.

### Quantification of 4MSOB-ITC and breakdown products with LC–MS

4MSOB-ITC and 4MSOB-amine were measured in the supernatants of bacterial cultures to confirm the successful knock-out of *saxA* in PsKO and to follow the degradation of the ITC over time. All supernatants were stored at −20°C before the measurement. Freshly prepared external standard curves of 4MSOB-ITC (L-sulforaphane, CAS 142825-10-3; Sigma Aldrich) and 4MSOB-amine (4-methanesulfinylbutan-1-amine; Enamine Germany GmbH, Frankfurt a.M., Germany) were measured together with the samples. The measurements were conducted as described in [28], and details are described in the Supplementary Methods.

### Transcriptomics

#### Growth of bacterial cultures

Overnight cultures were normalized to OD_600_ = 0.2 and mixed in equal amounts with either a partner strain (co-cultures) or R2A (monocultures). One hundred microlitres was used as inoculum for 1.9 ml R2A supplemented with 15 μg/ml 4MSOB-ITC or DMSO as a control. The cultures were incubated in 5-ml tubes on a shaker at 30°C, 150 rpm, for 17–18 h. We chose this late timepoint to evaluate the long-term effects of ITC exposure and SaxA-dependent rescue on bacterial gene expression and interactions. Monocultures and co-cultures were performed in separate experiments. The experimental design is visualized in Supplementary Fig. S2.

#### RNA extraction

Bacterial cultures were harvested by centrifugation at 5000×*g* for 20 min at 4°C. Supernatants were discarded and the cell pellets were frozen in liquid nitrogen. Total RNA was extracted using lysozyme and a hot phenol extraction protocol followed by a DNAse treatment, and details are described in the Supplementary Methods. All samples were sent to Eurofins Genomics (Konstanz, Germany) on dry ice, where the rRNA depletion, library preparation, and sequencing were performed.

#### Data analysis

Raw reads were trimmed using the implementation of trimmomatic in the GREP2 package in R [45] and were then mapped to the whole genomes of Pl and Ps [28] using Rsubread function align [46] and assigned to genes using the featureCounts function [47]. Low abundant genes were excluded from the subsequent analysis, and only genes with 10 reads in at least 3 samples were considered. The count data of the remaining genes were normalized and Log2FoldChanges (L2FC) were calculated using DESeq() function with default settings in the DESeq2 package which include a Wald test and Benjamini–Hochberg correction [48]. Using the ggplot2 package [49], a PCA was plotted of the top 500 genes with the highest variance using rlog-transformed data for the plotPCA function in DESeq2 package. To analyse differentially expressed genes (DEGs), the results of pairwise DESeq2 calculations were shrunk using lfcshrink function with apeglm [50]. After checking the dispersal in MA plots, the data were subset to keep only significant (*P* < .05) genes with |L2FC| >1 for the analysis. Venn diagrams with shared and unique DEGs between treatments were determined and visualized using the package VennDetail [51]. We summarized Pl’s reaction to the ITC by filtering for DEGs with |L2FC| >2 to prioritize important processes. To characterize the reaction of Ps or Pl to the partner strain, we generated gene to GO term maps for Pl and Ps with GO annotations of their whole genomes which were annotated using bakta [52]. Next, we tested for significant enrichment of GO terms using the function runTest with the classic algorithm followed by a Fisher test using the packages topGO [53]. For each pairwise comparison, significantly enriched GO terms were determined. The package VennDetail [51] was used to assess shared and unique terms between treatments. GO terms of each strain’s reaction to the partner with or without 4MSOB-ITC were plotted together.

## Results

### SaxA-mediated ITC degradation by the pathogen *P. viridiflava* (Ps) facilitates growth of the co-colonizing commensal *Plantibacter* (Pl)

To investigate a potential positive effect of SaxA-mediated 4MSOB-ITC degradation on ITC-sensitive commensals (Fig. 1A), we generated a knock-out of *saxA* (hereafter: PsKO) in our model pathogen strain *P. viridiflava* 3D9 (hereafter: Ps). We established earlier that the wildtype strain degrades 4MSOB-ITC to 4MSOB-amine [28]. Although homologs of ITC efflux pumps were identified in the genome of Ps [28], growth of PsKO was significantly inhibited by increasing 4MSOB-ITC concentrations at 30°C (Fig. 1B). The knock-out of *saxA* resulted in a complete loss-of-function, showing that SaxA in Ps is completely responsible for the degradation of 4MSOB-ITC (Fig. 1C). We used *Plantibacter* sp. 2H11-2 as a model ITC-sensitive commensal because it is clearly inhibited by 4MSOB-ITC in a concentration-dependent manner [28], it was isolated from the same population of wild *A. thaliana* plants as Ps, and it can be differentiated from Ps/PsKO based on colony morphology (Supplementary Fig. S1). To check the interaction between Ps or PsKO with Pl, we screened a wide range of ITC concentrations which are likely to occur in damaged and healthy leaves. First, we established a “public good assay” (Fig. 1D): Pl was poured into R2A agar together with different concentrations of 4MSOB-ITC, and Ps or PsKO was drop spotted on top of the agar. As expected, PsKO colonies were clearly inhibited by increasing ITC concentrations (Supplementary Fig. S3). At 0 and up to 40–45 μg/ml 4MSOB-ITC, Pl grew densely in the entire well (opaque agar) with both Ps and PSKO. At a high ITC concentration of 70–75 μg/ml, Pl growth was completely inhibited after 4 days (clear agar). At intermediate 4MSOB-ITC levels (55–65 μg/ml), however, we observed a zone of Pl colonies around the Ps but no or less around the PsKO colony (Fig. 1E, 1F, Supplementary Fig. S3). Taken together, Pl benefits from SaxA-mediated 4MSOB-ITC degradation and is able to grow in the vicinity of Ps.

### The public good effect of SaxA rescues Pl at high ITC concentrations in regular growth medium and leaf extract medium

**Figure 2:**
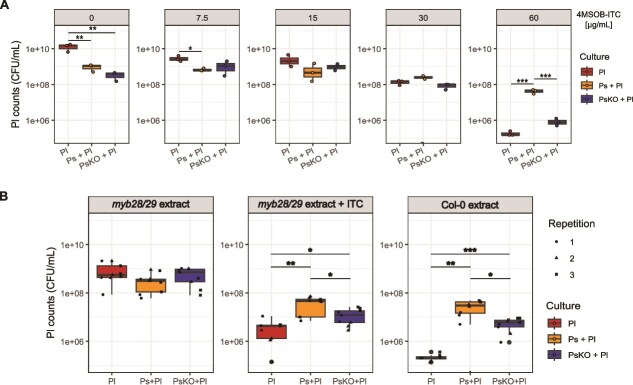
Benefit of Pl from *saxA*-mediated 4MSOB-ITC degradation by Ps after 22–24 h. (**A**) Bacterial counts of Pl in mono- and co-culture with Ps or PsKO after 22 h of incubation at 30°C in R2A broth supplemented with different 4MSOB-ITC concentrations (*n* = 3). Counts for Ps and PsKO are shown in Supplementary Fig. S4. *P*-values only for significant comparisons are shown (*t*-test, *P* < .05). (**B**) Bacterial counts of Pl in leaf extract medium without ITC (*myb28/29* extract), with 60 μg/ml 4MSOB-ITC (*myb28/29* extract + ITC) or with a mixture of naturally occurring GLS breakdown products (Col-0 extract, which has mainly 4MSOB-ITC) (*n* = 3, 3 independent repetitions with different batches of leaf extract medium). Counts for Ps and PsKO are shown in Supplementary Fig. S5. *P*-values only for significant comparisons are shown (*t*-test, fdr adjusted). Significant comparisons in A and B are shown as asterisks: ^*^P < .05, ^**^P < .01, ^***^P < .001.

Next, we checked whether Pl benefited from SaxA at a range of ITC concentrations after 22 h in liquid medium (Fig. 2A). As expected, Pl counts in monoculture were reduced with increasing ITC concentration, clearly evident after 30 μg/ml. When no or only little (7.5 μg/ml) 4MSOB-ITC was added, Pl grew better alone than with Ps or PsKO, likely due to higher nutrient availability in the absence of a competitor. Since Pl was not completely outcompeted in the presence of Ps, there is probably no direct inhibitory action from Ps towards the commensal. At low to intermediate concentrations (15 and 30 μg/ml), there were no significant differences between final bacterial counts in Pl monoculture and co-cultures. However, at the highest concentration (60 μg/ml), we clearly observed a public good effect whereby Ps with SaxA, but not PsKO, rescued Pl growth (*P* = .0003 Ps + Pl vs. Pl, *P* = .003 Ps + Pl vs. PsKO + Pl; Fig. 2A). Pl growth with PsKO was similar to Pl monoculture, suggesting that the ITC toxicity dominated and nutrient competition played only a minor role, if any, at this concentration (Fig. 2A). Ps was not affected by the presence of Pl, whereas, unexpectedly, PsKO was negatively affected by the presence of Pl at higher ITC concentrations (Supplementary Fig. S4).

Since Pl benefited the most at 60 μg/ml 4MSOB-ITC, we were interested to see how this translates to a more natural set-up where we expose mono- and co-cultures to plant metabolites which would be released in wounded leaves (Fig. 2B). For example, due to herbivory or pathogen attacks, aliphatic GLS are broken down and GLS breakdown products like ITCs are released from broken plant cells. To mimic this process, we crushed *A. thaliana* leaves in R2A broth (“leaf extract medium”) either from wildtype Col-0 (normal aliphatic GLS content) or mutant *myb28/29* leaves (no aliphatic GLS). Pl counts were significantly higher in co-culture with Ps than in monoculture in Col-0 extract medium (*P* = .0029 Pl vs. Ps + Pl, *P* = .0005 Pl vs. PsKO + Pl; Fig. 2B) as well as in *myb28/29* extract medium supplemented with 60 μg/ml 4MSOB-ITC (*P* = .0023 Pl vs. Ps + Pl, *P* = .0144 Pl vs. PsKO + Pl; Fig. 2B), but not in *myb28/29* extract medium with no ITC added. Contrary to the results in R2A broth, Pl also benefited from PsKO in this more complex leaf extract medium, but less than from Ps (Fig. 2B). Interestingly, Ps and PsKO growth both benefited from Pl in all three leaf extract media (Supplementary Fig. S5), an effect not observed in pure R2A medium (Supplementary Fig. S4).

As a preliminary test of the relevance of SaxA as a public good *in planta*, we wounded Col-0 and *myb28/29* leaves and inoculated them with Pl monoculture or co-cultures with Ps or PsKO. We did not see the expected pattern of Pl benefiting from Ps but not PsKO in Col-0 leaves (Supplementary Fig. S6). However, we did observe an effect of SaxA on Pl loads. Specifically, Pl reached about four times higher levels on *myb28/29* than on Col-0 in co-culture with Ps (*P* = .023), but not alone or with PsKO (Pl monoculture: *P* = .985; PsKO + Pl: *P* = .110). In addition, the difference between Pl alone and Pl with Ps was marginally relevant (*P* = .064) only on *myb28/29* but not on Col-0. Thus, our results are consistent with a SaxA public good effect *in planta* that is in this limited experimental set-up most likely due to non-aliphatic ITCs still present and induced in *myb28/29* (see discussion).

### Pl is transiently rescued by SaxA-mediated ITC degradation at lower ITC concentrations

**Figure 3:**
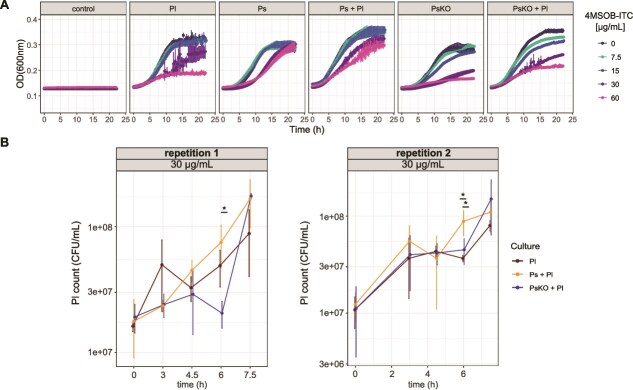
Benefit of Pl from *saxA*-mediated 4MSOB-ITC degradation by Ps over time. (**A**) Growth curves of mono- and co-cultures of Ps, Pl, and PsKO at 4MSOB-ITC concentrations from 0 to 60 μg/ml at 30°C (mean ± standard deviation, *n* = 3). The strains were inoculated with OD = 0.2, and the initial inoculum of the co-cultures was OD = 0.4. The OD_600_ was measured in 15-min intervals over the course of 24 h. Control wells were inoculated with sterile medium. (**B**) Bacterial counts of Ps, PsKO, and Pl in mono- and co-cultures over the course of 7.5 h (mean ± standard deviation, *n* = 3, 2 independent repetitions). Facets indicate the counted strain and the starting concentration of 4MSOB-ITC. Significant comparisons at all timepoints are shown as asterisks: ^*^P < .05, ^**^P < .01, ^***^P < .001 (*t*-test, fdr adjusted). Counts for Ps and PsKO are shown in Supplementary Fig. S8.

To check for transient growth benefits at lower ITC concentrations, in the next step we studied the growth dynamics over time of the individual Pl, Ps, and PsKO strains and their co-cultures in liquid medium supplemented with 4MSOB-ITC concentrations from 0 to 60 μg/ml (Fig. 3A). As observed earlier [28] and expected by the endpoint CFU counts (Fig. 2A), Pl started to be affected at 15 μg/ml in monoculture and was clearly reduced by 30 μg/ml. Its growth slowed further to only about one-third of the original OD_600_ at 60 μg/ml 4MSOB-ITC (Fig. 3A). ITC degradation by Ps was already evident after 3–6 h (Supplementary Fig. S7). Thus, we looked for transient effects at 0–7.5 h at 30 μg/ml 4MSOB-ITC because Pl was already inhibited at this concentration early in the time course when grown alone (Fig. 3A). Indeed, we observed a public good effect in the Ps + Pl co-culture after 6 h (Fig. 3B, repetition 1: *P* = .023, repetition 2: *P* = .029 for Ps + Pl vs. PsKO + Pl). However, this effect disappeared by 7.5 h (Fig. 3B) indicating only a transient effect on Pl growth at intermediate ITC levels. However, at 30 μg/ml ITC, Ps also benefited from Pl for a short time between 12.5 and 15 h independent of the ITC content (Supplementary Fig. S9), indicating possible mutualistic benefits rather than Pl simply exploiting Ps degradation of the toxin. Taken together, Pl growth in monoculture was temporarily inhibited by intermediate ITC levels (30 μg/ml) and completely inhibited after 22–23 h at higher ITC concentrations (60 μg/ml), but with Ps it can overcome this growth inhibition in R2A broth and leaf extract medium.

### SaxA prevents strong Pl gene expression changes at low 4MSOB-ITC levels

Low ITC concentrations are likely to be encountered by bacteria even in healthy leaf tissue [27] where they may not exert significant cytotoxic or growth inhibiting effects [30, 31, 54] but act on a transcriptional level. In accordance, we did not observe significant growth reduction of Pl when supplemented with 15 μg/ml 4MSOB-ITC (Fig. 2A). To investigate the effects of low 4MSOB-ITC levels on the long-term interaction between Ps and Pl, we grew them either alone or together supplemented with 15 μg/ml 4MSOB-ITC and evaluated their gene expression profiles using RNA sequencing after 17–18 h.

**Figure 4:**
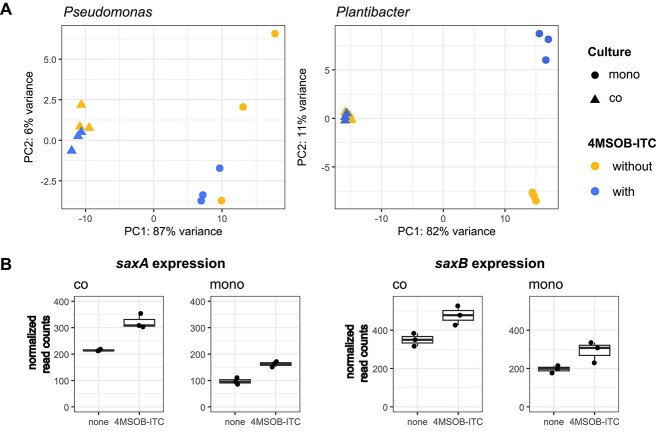
ITC effects on Ps and Pl gene expression, especially *sax* expression. (**A**) Comparison of partner and ITC effect on both isolates. PCA of rlog-transformed DESeq results. The partner effect on Ps and Pl reads is correlated to a higher proportion of the variance than the ITC effect. Triangles show co-cultures, and circles show monocultures of Ps (left panel) and Pl (right panel) with or without 4MSOB-ITC (*n* = 3 per condition). (**B**) Significant upregulation of *saxA* and *saxB* in Ps mono- and co-culture in response to 4MSOB-ITC exposure. Read counts of *saxA* and *saxB* of transcriptomics experiment were normalized using DESeq and plotted. Both genes were significantly differently expressed in ITC-treated and non-treated samples (*n* = 3) in mono- and co-cultures (see Supplementary Table S3 for Log2FoldChanges and adjusted *P*-values).

Because Ps protects itself by both detoxification and metabolite export, as expected, its transcriptome was hardly affected by 4MSOB-ITC (Fig. 4A). We detected only three significant DEGs in Ps (log_2_fold-change |L2FC| > 1, *P* < .05) in response to the ITC in monoculture or co-culture (Supplementary File with all DEGs in all comparisons). At the sampling timepoint, Ps had already been growing without the ITC for 12–15 h due to the rapid rate of degradation. Even though the ITC was no longer present, both *saxA* and *saxB* genes were still slightly but significantly upregulated in response to 4MSOB-ITC in both Ps mono- and co-cultures (Fig. 4B, Supplementary Table S3; *saxA*: L2FC = 0.5238, *P*_adj_ = .0012 (mono); L2FC = 0.6296, *P*_adj_ = .0057 (co), *saxB*: L2FC = 0.3690, *P*_adj_ = .0217 (mono); L2FC = 0.4415, *P*_adj_ = .0451 (co)). The transcriptional regulator *saxC* was not upregulated upon ITC exposure (Supplementary Table S3), and efflux pumps of type “Multidrug efflux pump subunit AcrB” with COG0841 number which were earlier associated with *saxF* [11, 55] were not significantly differentially expressed either (Supplementary Table S4), indicating ITC degradation rather than ITC efflux to be the major resistance mechanism in this strain.

In contrast to Ps, Pl was more affected by the low levels of 4MSOB-ITC. In monoculture, we detected 100 DEGs (|L2FC| > 1, *P*_adj_ < .05, Supplementary File with all DEGs, Supplementary Fig. S10) and transcriptomes of Pl monocultures with or without ITC clearly separated in the PCA (Fig. 4A). A more stringent filtering (|L2FC > 2|) left only 17 DEGs (Supplementary Table S5). Of these, five were downregulated, and their predicted protein annotations suggest links to costly metabolic processes. Among the 12 upregulated DEGs, two were annotated with proteins which can be involved in oxidative stress responses in other bacteria (ACEBMG_0398, nitroreductase; ACEBMG_15290, NADPH:quinone reductase) [56, 57] and another three were annotated as transcriptional regulators (ACEBMG_16910, HTH-type transcriptional regulator CmtR; ACEBMG_17225, HxlR family transcriptional regulator; ACEBMG_03025, AraC family transcriptional regulator) (Supplementary Table S5). Taken together, Pl displays a general transcriptional reprogramming after exposure to 4MSOB-ITC with increased defence against oxidative stress and reduced metabolism possibly to save energy. None of these effects were observed when Pl was co-cultured with Ps (Fig. 4A) where no DEGs were identified (*P*_adj_ < 0.05, |L2FC| > 1), indicating that the Ps SaxA activity fully protected Pl from ITC toxicity at low 4MSOB-ITC levels.

### Co-cultivation with Pl reduces virulence expression in Ps independent of 4MSOB-ITC

**Figure 5:**
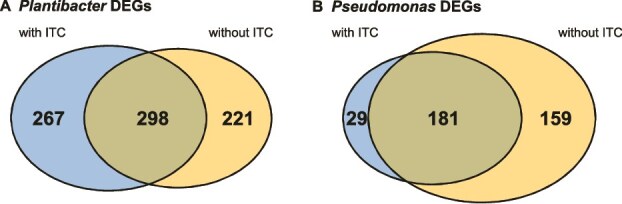
Effect on Pl and Ps on gene expression in each other in relation to previous 4MSOB-ITC exposure. (**A**) Pl reacted to Ps and significantly changed the regulation of 267 genes (L2FC > 1, *P* < .05) when the co-culture was exposed to 4MSOB-ITC, or 211 genes without ITC. 298 DEGs represent Pl’s reaction to Ps independently of 4MSOB-ITC. (**B**) Ps reacts to Pl with 181 DEGs which are independent of previous ITC exposure. This strain regulates more unique DEGs (159) without exposure to 4MSOB-ITC than after exposure (29 DEGs). All DEGs of all treatments are provided in the supplementary file.

Next, we evaluated whether the effect of the Pl or Ps strain on each other went beyond ITC (Fig. 4, Fig. 5A, 5B, all DEGs of all treatments are provided in the Supplementary File). Interestingly, the reaction of each strain to the other was at least partly influenced by previous ITC exposure even though we only saw a strong ITC effect on Pl monocultures where it was not degraded by Ps. There were 565 and 519 DEGs identified in Pl in response to Ps in the presence and absence of 4MSOB-ITC, respectively. More than half of these DEGs were independent of previous ITC exposure (298 DEGs, Fig. 5A). For Ps it was similar, with 210 or 340 DEGs found in response to Pl when the ITC was added or not, respectively. A high fraction (181 DEGs) was independent of 4MSOB-ITC, but without ITC Ps differentially regulated an additional 159 genes in response to Pl. In the presence of ITC, Ps uniquely differentially expressed only 29 genes (Fig. 5B), suggesting that Ps responded more to Pl when there was no need to degrade 4MSOB-ITC.

**Figure 6:**
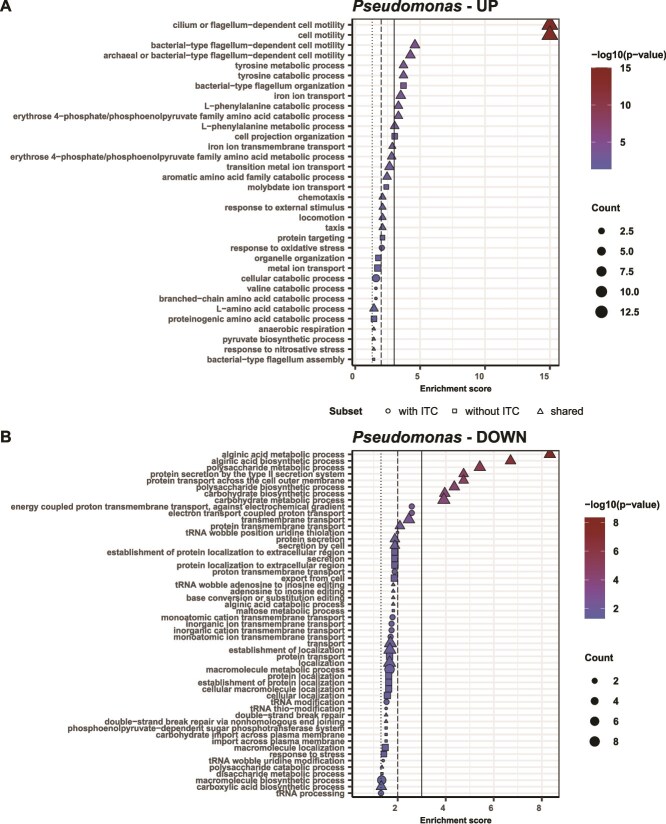
GO term enrichment analysis for the reaction of Ps to Pl with or without ITC. Significantly enriched GO terms for upregulated (**A**) and downregulated (**B**) Ps genes in reaction to Pl. Shared and unique genes, *P*-values (Fisher’s significance test), and the count number of GO terms for each category are indicated. Horizontal lines indicate significance levels: *P* = .001 (straight line), *P* = .01 (dashed line), *P* = .05 (dotted line). GO term enrichment analysis for the reaction of Pl to Ps with or without ITC is shown in Supplementary Fig. S10.

In line with this, most processes that were significantly enriched in our GO term analysis were independent of ITC exposure. The most significant (*P* < .01, Fisher test) GO terms for upregulated DEGs by Ps in response to Pl (Fig. 6A) were linked to flagellum-dependent motility, chemotaxis and locomotion, catabolism of diverse amino acids (especially Phe, Tyr, Val, aromatic and branched amino acids) and iron transport, all independent of the ITC. Without 4MSOB-ITC, Ps in addition upregulated transport of ions like molybdate, while with 4MSOB-ITC, Ps upregulated oxidative stress responses. The downregulated GO terms (Fig. 6B) for Ps were associated with transmembrane transport processes and processes linked to general protein localization and protein secretion, as well as features associated with virulence and biofilm formation in plant pathogenic pseudomonads [58] such as biosynthesis of alginate, polysaccharides, and carbohydrates. Additionally, protein secretion by the type II secretion system which had been associated with virulence in *Xanthomonas* sp. [59] was downregulated as well. All these processes were independent of the ITC. Without 4MSOB-ITC, Ps downregulated processes linked to protein localization and secretion as well; with 4MSOB-ITC, it additionally downregulated energy/electron coupled transmembrane transport and ion transmembrane transport processes. The ITC-independent changes suggest a shift in Ps lifestyle from cell aggregates/biofilms to more motile growth in reaction to Pl.

At the same time, the GO processes upregulated by Pl in reaction to Ps all were independent of 4MSOB-ITC. These included catabolic processes related to amino acids, carboxylic acids, and organic acids, as well as translation (Supplementary Fig. S11, Supplementary File). Pl reacted to Ps by downregulating processes associated with GO terms on transport (metal ions, especially cobalt, magnesium, potassium) which was independent of 4MSOB-ITC. With ITC, Pl additionally decreased carbohydrate transport processes, and without ITC, Pl additionally decreased processes relevant for ion homeostasis (Supplementary Fig. S11, Supplementary File).

In conclusion, the reaction of both strains to each other was mostly independent of the presence of 4MSOB-ITC, likely because of rapid ITC degradation by Ps in co-cultures but suggests that the interaction of Ps with a commensal can shape its virulence.

## Discussion

In this study, we showed that plant metabolites can modulate interactions between plant commensal and pathogenic bacteria. Specifically, detoxification of antimicrobial plant metabolites by one leaf bacterium will directly benefit other sensitive bacteria in its neighbourhood. This public good effect of toxin degradation has so far only been studied in antibiotic degraders [60–62]. Enzymes that degrade antibiotics or catalyse reactions that make them non-toxic can confer a benefit on susceptible co-colonizing bacterial strains [63]. This effect is also called “passive resistance” and has been especially studied in connection with β-lactamases that degrade antibiotics like ampicillin or penicillin [60, 63, 64]. Here, we extend this principle to the metabolism of the plant-derived antimicrobial ITCs. This mechanism may not just be relevant in leaves as proposed here using leaf bacterial isolates, but it may also play roles in other environments where microbes face ITCs like in soils and insect guts [8, 15].

**Figure 7:**
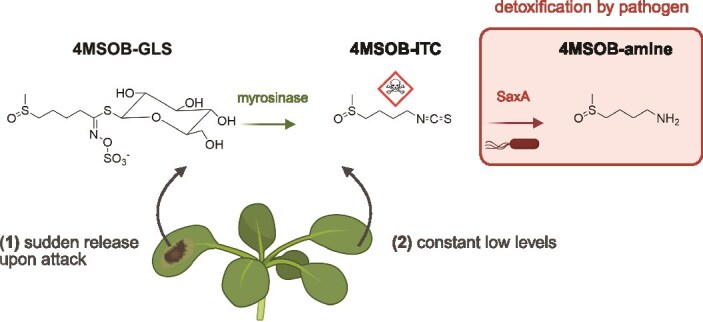
Microbial leaf colonizers are exposed to ITCs. Bacteria are expected to face different ITC concentrations in plant leaves ranging from high ITC concentrations (1) when glucosinolates (GLS) are converted to antimicrobial isothiocyanates (ITCs) by myrosinases either in necrotic leaf tissue upon attack by a plant pathogen or when insects bite into a leaf to low, sublethal concentrations (2) when GLS are constantly turned over to ITCs (and cysteine) or when GLS are broken down by co-colonizing microbes. Depending on the micrometre distance between ITC release and bacterial cells, they may be also exposed to a range of intermediate ITC levels. Plant pathogens like *Pseudomonas viridiflava* 3D9 detoxify ITCs by the ITC hydrolase SaxA to the corresponding amine. The figure shows 4-methylsulfinylbutyl glucosinolate (4MSOB-GLS) which is the major GLS in *A. thaliana* Col-0 and its breakdown products. Created in BioRender. Unger, K. (2026) https://BioRender.com/7ox0x2a.

In the specific case of ITCs in leaves, studied here, there is likely a range of ITC concentrations at which ITC detoxification by SaxA might become relevant for a commensal like *Plantibacter* sp. (Fig. 7). First, when a plant is under attack by a herbivore or pathogen, it may suddenly and locally release high ITC concentrations [25, 26]. Second, ITCs may be present in low concentrations in leaf tissue due to constant degradation of GLS to ITCs for sulphur recycling to cysteine [27] or when GLS-utilizing bacteria form ITCs from GLS present in the plant or on the plant surface [28]. Unfortunately, studies measuring ITC concentrations in living plant leaves are rare. Wang *et al*. [30] measured about 42 μM (=7.4 μg/ml) 4MSOB-ITC in bulk samples of apoplastic fluid of non-infected *A. thaliana* Col-0 leaves, and we and others [28, 65] measured glucosinolates on the leaf surface which can be broken down by some leaf colonizers [28] and likely expose co-colonizing strains to ITCs. Upon infection and lesion formation, an increase of ITCs at the site of infection is expected. For example, an increase from 17.7 μg ITC/g dry leaf weight to 88.6 μg/g dry weight was measured in whole leaves which were infected with the fungus *Sclerotinia sclerotiorum* [6]. Assuming ITC levels increase similarly in the apoplast, we expect at least 37 μg/ml in such a case, and probably concentrations are even higher depending on how close bacterial cells are to the lesion sites. ITCs likely dissipate away from the site of production; thus, we expect microbes in leaves to be exposed to a wide range of ITC concentrations. In addition, growth in biofilms or cell aggregates [66, 67], patchy distribution of microbes [66], and non-uniform distribution of metabolites in leaves [65, 68] make it hard to estimate the exposure of individual bacterial cells to 4MSOB-ITC. We used leaf extract medium produced from crushed leaves of *A. thaliana* wildtype Col-0 to mimic the metabolic landscape to which leaf microbes might be exposed during herbivore or pathogen attack. This approach allowed us to focus on GLS breakdown products independent of other plant defences that likely arise during Ps virulence. Growth of Pl in medium produced from Col-0 leaves was comparable to medium from aliphatic GLS-free *myb28/29* leaves supplemented with 60 μg/ml 4MSOB-ITC. This suggests that 60 μg/ml might be a good approximation for long-term effects of this ITC on Ps–Pl interactions in wounded leaves. Our findings illustrate the need to more precisely quantify bacterial exposure to ITCs and other specialized metabolites in living plant tissues, ideally in a spatially resolved manner.

We hypothesize that in plants, the sudden release of high ITC levels (Fig. 7, first scenario) presents a serious threat for the fitness of ITC-sensitive commensals because ITCs severely impair cell membranes and non-specifically bind to proteins, affecting their activity [33, 34]. Reductions in the populations of specific commensal bacteria might alter the balance of the entire leaf microbiome, which is of great importance because they are needed to help protect the plant from disease [36, 69]. Constant low ITC levels in leaves (Fig. 7, second scenario) might not directly inhibit bacterial growth but still could alter transcriptional activity of commensals. In our study, Pl growth was barely inhibited by 15 μg/ml 4MSOB-ITC, but its transcriptome already changed strongly at these low levels. We found several transcriptional regulators to be more expressed when exposed to the ITC, suggesting a general transcriptional reprogramming. Additionally, Pl upregulated genes which were associated with functions in oxidative stress response in other bacteria [56, 57]. This agrees with other studies showing effects of sublethal ITC concentrations on bacterial activity like induction of stringent response [70], inhibition of quorum sensing [54], or suppression of virulence [30, 31]. In co-culture, Ps degraded the ITC and so Pl’s transcriptome was not affected, demonstrating a clear public good effect even at low ITC concentrations. Nevertheless, previous ITC exposure influenced how Ps and Pl reacted to one another even when the ITC had already been degraded hours before. Thus, changes in the transcriptome may cause subsequent changes in microbial interactions.

Based on our results, we propose that SaxA, which is known to play important roles for virulence in different pathosystems [6, 30, 32], may indirectly benefit plant health by rescuing commensal or plant-beneficial bacteria. However, our study is mainly based on *in vitro* experiments to focus on the effect of the metabolite 4MSOB-ITC and to be able to link effects to SaxA-mediated ITC degradation under controlled conditions. These are different from the situation e.g. in the leaf apoplast, where the chemical landscape, especially during wounding, would contain diverse plant-derived primary and secondary metabolites [71]. Some of these, like ITCs derived from non-aliphatic GLS, can also be detoxified by SaxA [32], but metabolites like camalexin that also accumulate would not. This more complex picture fits to what we observed in the *in planta* experiment, where SaxA had a public good effect in the aliphatic GLS-free *myb28/29* mutant. Because SaxA has no known functions beyond its role as an ITC hydrolase, it is likely that in the *myb28/29* background indole or other ITCs upregulated after wounding played a role. This also fits to our previous observation with the fungal pathogen *Sclerotinia sclerotiorum*, where fungal SaxA affected the microbiome in necrotic fungal lesions in the *myb28/29* background [13]. A more detailed approach will be needed to understand the effect of the complex and diverse defence chemistry *in planta*. Although it is clear in well-mixed liquid cultures that SaxA can function as a public good for aliphatic ITC degradation, we did not observe this effect *in planta* in Col-0. There are many possible reasons why this was not observed. For one, many variables such as infection path, incubation and sampling times, and environmental conditions may need optimization. Additionally, in living leaves, pattern-triggered immunity and effector-triggered immunity play a major role in controlling bacterial and pathogen growth and could also interact with defence chemistry in unpredictable ways to influence public good dynamics [72]. Our study did not investigate the interplay of defence metabolites like 4MSOB-ITC with other components of the immune system which may as well influence microbial interactions with relevance for plant health*.* Taken together, it is necessary for future work to better understand exactly how SaxA functions as a public good *in planta*.

Bacterial strains that do not pay the cost of a public good often gain an advantage and may eventually outcompete the producer of the public good, known as the “tragedy of the commons” [73]. We expected to observe that Pl would come to dominate the co-culture because it benefited from SaxA-mediated ITC degradation without having to invest energy. However, we only observed this effect transiently at 6 h at intermediate ITC concentrations (30 μg/ml). In the long run, neither Ps nor Pl outcompeted the other strain. Instead, our experiments demonstrate a clear stepwise benefit for Pl from Ps with increasing ITC concentration until Pl was fully dependent on Ps at 60 μg/ml 4MSOB-ITC. Interestingly, Pl also benefited from PsKO in leaf extract medium, suggesting interactions independent of ITC degradation by SaxA. In return, we also observed a transient benefit for Ps and PsKO in leaf extract medium which was also independent of the ITC. These findings might indicate possible mutualistic benefits between both strains that can stabilize an interaction [74, 75] and therefore may provide an explanation why Ps did not outcompete Pl. At low ITC concentrations (15 μg/ml), each strain upregulated amino acid catabolism in reaction to the other, which may be a sign of competition. This fits previous studies that suggested substrate competition to be an important driver of leaf bacterial interactions [17, 76]. However, the upregulation of amino acid catabolism in both strains might also be an indication of cross-feeding, but we cannot exclude or confirm this possibility without further experiments. Cross-feeding could also be supported by the release of 4MSOB-amine which would likely only be relevant under nitrogen-deficient conditions. Together, we found suggestions for both cooperation and competition between Ps and Pl and the outcome of their interaction was highly dependent on the 4MSOB-ITC concentration.

In the absence of ITCs, we observed a clear change in the lifestyle of Ps when it was co-cultured with Pl. Ps increased its motility and decreased biofilm-related transcriptional processes like alginate and polysaccharide production, and protein secretion by the type II secretion system. Alginate production is a major virulence factor of plant pathogenic pseudomonads [58], and the type II secretion system plays a role for virulence of opportunistic *Xanthomonas* sp. [59]. Thus, co-colonization with a commensal like Pl could confer disease protection to the host plant by suppressing these processes. Interestingly, the reaction of Ps to Pl has similarities to the transcriptome of pathogenic *P. syringae* during its epiphytic non-virulent life stage which is characterized by flagellar motility, chemotaxis, and higher activity of amino acid catabolism than in its endophytic stage [77]. Thus, these changes could be a general switch towards a more commensal lifestyle, which is well known to be common for *P. viridiflava* in wild *A. thaliana* populations [78]. Moreover, since virulence of Ps relies on nutrients such as amino acids [79] and sugars [80], the utilization of these nutrients by Pl may represent another way to suppress the virulence of Ps. In conclusion, another side effect of Ps rescuing Pl by ITC detoxification might be the suppression of Ps virulence and a switch towards a more commensal lifestyle. Understanding how these virulence changes affect microbial interactions in the leaf will require future studies *in planta*.

Detoxification of plant specialized metabolites, such as 4MSOB-ITC, may hence be highly relevant in plant–microbe–microbe interactions and likely also in other environments like insect guts or in soil. Further *in planta* experiments on small sampling scales with longer incubation times and controlled ITC exposure will show whether the suggested possibilities of how the Ps virulence factor SaxA may indirectly contribute to plant health by rescuing commensals like Pl holds true in living plant leaves. This knowledge contributes to a more holistic understanding of the microbial interactions occurring with significant implications for plant resistance to pathogens and may also contribute to the development of new biocontrol agents.

## Supplementary Material

Supplementary_Material_ycag004

## Data Availability

Raw data and R scripts to generate the figures are publicly available on figshare (https://figshare.com/projects/Pseudomonas_virulence_factor_SaxA_is_a_public_good_that_protects_a_commensal_plant_bacterium_from_a_glucosinolate-derived_host_defence_metabolite/242066). The raw RNA-seq data generated in this study have been deposited in the NCBI-SRA database under accession code: PRJNA1405973.
