# Supplementary material for: Pseudomonas virulence factor SaxA detoxifies plant glucosinolate hydrolysis products, rescuing a commensal that suppresses virulence gene expression
Source: ISME Commun. 2026 Jan 9;6(1):ycag004. doi: 10.1093/ismeco/ycag004 (PMC12903958; doi:10.1093/ismeco/ycag004)
Supplement: Supplementary_Material_ycag004 [file supplementary_material_ycag004.zip › Supplementary Material_SaxA-Ps+Pl_FINAL.pdf]

Supplementary Material for:

Pseudomonas virulence factor SaxA detoxifies plant glucosinolate hydrolysis products, rescuing a commensal that suppresses virulence gene expression

Kerstin Unger[1], Rebecca Ruiter[1], Michael Reichelt[2], Jonathan Gershenzon[2], Matthew T. Agler[1,3]*

[1] Plant Microbiosis Group, Institute of Microbiology, Friedrich Schiller University Jena, Jena, Germany.
[2] Department of Biochemistry, Max-Planck-Institute for Chemical Ecology, Jena, Germany.
[3] Cluster of Excellence Balance of the Microverse, Friedrich Schiller University Jena, Jena, Germany.

*Corresponding author:

Matthew T. Agler
Institute of Microbiology, Plant Microbiosis Group
Friedrich Schiller University Jena
Neugasse 23
07743 Jena, Germany
E-mail: matthew.agler@uni-jena.de
Tel: +49 (0)3641 9 49980

Table of contents:

Supplementary Methods

Supplementary Tables 1-5

Supplementary Figures 1-11

Supplementary References

Supplementary Methods

Knock-out of *saxA* in *Pseudomonas viridiflava* 3D9

First upstream (UP) and downstream (DW) regions with an overlap of 78 nt and 84 nt with *saxA* were amplified: 5 µL 5x Q5 Reaction Buffer, 0.5 µL 10 mM dNTPs, 1.25 µL 10 µM upA_fwd / downA_fwd, 1.25 µL 10 µM upA_rvs / downA_rvs, 0.25 µL Q5 High-Fidelity DNA Polymerase, 15.75 µL NFW, 1 µL gDNA. Initial denaturation: 98°C for 30 s, 30 cycles: 98°C for 10 s, 65 °C for 5 s, 72°C for 15 s, final extension: 72°C for 2 min, hold at 10°C. Both fragments were cleaned up using the QIAquick PCR & Gel Clean UP Kit (QIAGEN) and eluted in 30 µL 10 mM Tris-HCl pH=8. The UP fragment is 470 nt long and DW fragment is 483 nt long, and this strategy would result in a truncation of 729 nt in the gene *saxA*.

The pEXG2 plasmid was linearized: 20 µL pEXG2 (~1µg), 5 µL 10x rCutSmart Buffer, 1 µL EcoRI-HF (New England Biolabs), 1 µL HindIII-HF (New England Biolabs) and 23 µL NFW were mixed, digested for 60 min at 37°C, and inactivated at 80°C for 20 min. Five 50 µL reactions were combined and 700 µL 100% molecular grade ethanol with 25 µL sodium acetate (3M, pH 6.5) was added to precipitate the linear pEXG2 at -20°C for 4 h. To collect the plasmid, it was centrifuged at 20,000 x g for 40 min, washed with 70% ethanol and resuspended in 30 µL 10 mM Tris-HCl pH=8. To assemble the plasmid the Gibson Assembly Mastermix (New England Biolabs) was used and the plasmid and UP and DW regions were mixed 1:2 (molar concentrations). The reaction was incubated at 50°C for 60 min and then stored at 10°C until the transformation.

Chemically competent *E. coli* DH5α cells thawed on ice, 2 µL of the assembled plasmid was added and cells incubated for 30 min on ice. Next, they were heat shocked by exposing them to 42°C for 60 s. After another 5 min on ice, 900 µL LB (10 g/L tryptone, 5 g/L yeast extract, 10 g/L sodium chloride, pH = 7.0±0.2) medium was added followed by an incubation at 37°C, 200 rpm, for 1.5 h. The cells were centrifuged at 3500 x g for 5 min, the supernatant was discarded, and the cells were gently resuspended in 150 µL fresh LB broth which were plated on LB agar supplemented with 15 µg/mL gentamicin (Gent). After 48 h of incubation at 37°C positive colonies were picked and checked for the presence of the correctly assembled plasmid: cell material from individual colonies was resuspended in 0.05 M NaOH and boiled at 95°C for 15 min to extract crude DNA which was used for the subsequent PCR using 8pEX_fwd and 8pEX_rvs primers: 5 µL 5x Q5 reaction buffer, 0.5 µL 10 mM dNTPs, 1.25 µL 10 µM 8pEX_fwd, 1.25 µL 10 µM 8pEX_rvs, 0.25 µL Q5 High Fidelity DNA Polymerase, 15.75 µL NFW, 1 µL template. Initial denaturation: 98°C for 30 s, 30 cycles: 98°C for 10 s, 65 °C for 10 s, 72°C for 25 s, final extension: 72°C for 2 min, hold at 10°C. Using the QIAGEN Miniprep plasmid kit pEXG2-A plasmid was extracted from overnight cultures of *E. coli* DH5α-pEXG2-A in LB+Gent.

Using the same heat shock protocol as mentioned before, chemically competent *E. coli* ST18 cells were transformed with the plasmid and plated on LB plates containing gentamicin and 5-aminolevulinic acid (ALA). After 48 h of incubation at 37°C colonies were screened for the presence of the correct insert using 8pEX_fwd and 8pEX_rvs primers. 2 mL of an overnight culture of *E. coli* ST18-pEXG2-A and 2

mL of *Pseudomonas viridiflava* 3D9 overnight culture were used to inoculate 30 mL of fresh LB+Gent+ALA and LB, respectively. Both were incubated at 200 rpm for 2.5 h at 37°C and 30°C, respectively. Cells were harvested by centrifugation (3,500 x g, 5 min), washed with PBS and finally resuspended in 20 mL PBS. The OD600 was normalized to 0.5 for *E. coli* and 1.0 for *Pseudomonas viridiflava.* For the conjugation the strains were mixed in 3:1, 1:1 and 1:3 ratios (v/v), centrifuged again to recover the cell pellets and resuspended in 100 µL PBS. About 50 µL per mating spot were plated onto LB+ALA plates which were incubated at 30°C. On the next day, bacterial cells were resuspended in 800 µL fresh PBS and 10 µL, 100 µL and 500 µL of the suspensions were plated on LB+Gent plates to select against *E. coli* ST18 and for *Pseudomonas viridiflava* transformants. The transformants were streaked on fresh LB+Gent plates and tested for the absence of *E. coli* contamination (*uidA* primers [1]), presence of pEXG2-A plasmid with correct inserts (8pEX primers, Supplementary Table 1). For the counterselection positive colonies were cultured on LB supplemented with 10% sucrose at 30°C overnight.

Finally, the successful homologous recombination and thus deletion of *saxA* was checked using 9gen primers (Supplementary Table 1) which bind to the flanking regions of *saxA* in the genome of 3D9. Mastermix: 1.5 µL 10x Buffer B, 0.6 µL 10 mM dNTPs, 0.3 µL 10 µM 9gen_fwd, 0.3 10 µM 9gen_rvs, 1.5 µL 25 mM MgCl2, 9.65 µL NFW, 0.15 µL Taq Polymerase (Biodeal, Markkleeberg, Germany). Initial denaturation: 95°C for 2 min, 30 cycles: 95°C for 0:30 min, 61°C for 0:30 min, 72°C for 1:30 min, final elongation: 72°C for 5:00 min, cooling to 10°C. The product was sequenced (Eurofins Genomics) and to confirm the loss of function, 4MSOB-ITC and 4MSOB-amine were quantified in the supernatant of an overnight culture.

Details on *in planta* experiments

Wildtype Col-0 and aliphatic GLS-free mutant plants were grown in a gnotobiotic hydroponic system. The basic set-up was adapted from a flow-pot system [2] with 50 mL polypropylene syringes which were cut at the 20 mL mark as pots. Each pot was equipped with a 10 cm long jute yarn which was run through the syringe's outlet to function as a wick for the nutrient solution. A layer of "coarse" (particle size: 1.54 – 2.12 mm) perlite topped with a layer of "fine" (particle size: 0.635 – 1.54 mm) perlite (Perligran Premium) were filled in the pots and 10 pots were placed in one 5-liter microbox (SacO_2, Deinze, Belgium). The microboxes were autoclaved twice at 121°C for 20 min. Afterwards, the pots was soaked with about 300 mL of ½ Hoagland's solution, pH=6 [3]. Plants were surface-sterilised using 2 % bleach and 80 % ethanol, then they were vernalized in 0.1 % agarose at 4°C in the dark for four days. 3-5 seeds were sown per pot and plants were grown in a climate chamber (PolyKlima, Dinkelscherben, Germany) at 22°C/18°C, 16h/8h, day/night cycle at 75% light intensity. After one week the seedlings were thinned out to 2 seedlings per pot. After three weeks the seedlings were inoculated with Pl, Ps+Pl or PsKO+Pl mono- and co-cultures. First, three leaves per rosette were mechanically wounded by cutting from the margins to the midvein with sterile scissors as this was described to induce

the biosynthesis of GLS [4]. To produce the bacterial inoculum, Ps, PsKO and Pl cultures were grown overnight in R2A at 30°C, shaking at 150 rpm. After washing three times with 1x PBS, the OD_{600} was adjusted to 0.04 and Pl was mixed with Ps or PsKO or an equal volume of PBS to generate the mono- and co-culture inocula with $OD_{600} = 0.02$ of each strain. Each pot with two plants was inoculated by spraying from above with an airbrush system (two sprays for half a second each, ~70 µL/pot). 24 h after inoculation the whole rosettes were sampled because the leaves were too small to sample only the wounded leaves. The fresh weight was determined, and the plant material was crushed in a bead beater for 30 s at 1500 rpm. 10-fold dilution series were prepared in 1x PBS and two 5 µL droplets per sample were plated on R2A (Ps, PsKO counts after 24 h) or R2A supplemented with 1.5 µg/mL kanamycin (Pl counts after 48 h) and incubated at 30°C.

<u>Details on 4MSOB-ITC and 4MSOB-amine quantification with LC-MS</u>

4MSOB-ITC and its breakdown product 4MSOB-amine in bacterial cultures (n = 3) and non-inoculated medium controls (n = 3) were analyzed on an Agilent 1200 HPLC system (Agilent, Santa Clara, CA, United States) coupled to an API3200 tandem mass spectrometer (AB SCIEX, Darmstadt, Germany). The compounds were separated on an Agilent XDB-C18 column (5 cm × 4.6 mm, 1.8 µm, Agilent, Waldbronn, Germany). The mobile phase consisted of 0.05% (v/v) formic acid in ultrapure water as solvent A and acetonitrile as solvent B, at a flow rate of 1.1 mL/min. The elution gradient was: 0-0.5 min, 3-15% B; 0.5-2.5 min, 15-85% B; 2.5-2.52 min, 85-100% B; 2.25-3.5 min, 100% B; 3.5-3.51 min, 100-3% B, 3.51-6 min, 3% B. The ion spray voltage was maintained at 5500 eV in positive mode. The turbo gas temperature was set to 500 °C, nebulizing gas to 60 psi, drying gas to 60 psi, curtain gas to 35 psi, and collision gas to 3 psi. Details of multiple reaction monitoring (MRM) can be found in Supplementary Tab. 2. Analyst Software 1.6 Build 3773 (AB SCIEX) was used to acquire and process the data.

<u>Details on RNA extraction</u>

The frozen bacterial cell pellets were resuspended in 600 µL lysozyme solution (0.5 mg/ml in TE Buffer, pH=8) and transferred into a 2 mL tube. Samples with the gram-positive Pl were incubated for 5 min at 37°C before adding 60 µL of 10 % SDS. SDS was immediately added to all other samples with only gram-negative Ps. The samples were mixed by inverting the tube and incubated at 64°C for 1-2 min before 66 µL 1 M sodium acetate (pH= 5.2) were added. Next, 750 µL Roti-Aqua-Phenol (Carl Roth, Germany) was added and the samples were incubated at 64°C with constant shaking for 6 min. After 1 min on ice, the samples were centrifuged at 20,000 x g for 25 min at 4°C to separate the phases. The aqueous layer was transferred into phase-lock-tubes (5PRIME Phase Lock Gel heavy; Quantabio, Beverly, Massachusetts, USA) and 750 µL chloroform was added. Then, the samples were shaken for 10 s and incubated for 2-5 min at room temperature. For phase-separation, they were centrifuged at 20,000 x g for 10 min at 4 °C. The aqueous layer was transferred into a new tube and 1.4 mL of a 30:1 mix (ethanol 100 % : 3 M sodium acetate pH=6.5) were added. To recover the RNA/DNA pellet after an overnight incubation at -20°C, the samples were centrifuged at 20,000 x g for 30 min at 4°C. The pellet was washed with 80 % molecular grade ethanol, air-dried and eluted in 30 µL nuclease-free water (NFW) by shaking incubation at 66°C for 5 min. 10-15 µg RNA/DNA were incubated with 5 µL RDD

buffer and 1.25 µL DNAse I (QIAGEN) for 10 min at 25°C. The volume was filled up to 100 µL with NFW and 100 µL phenol-chloroform-isoamyl alcohol (25:24:1, Carl Roth) was added into the phase-lock-tube. The samples were shaken for ca. 15 s and incubated at room temperature for 2-5 min. Using another phase-lock-tube the phases were separated after centrifugation at 20,000 x g for 15 min at 12°C. The upper aqueous phase was transferred into a new tube and 300 µL of 30:1 ethanol sodium acetate mix was added to precipitate the remaining RNA overnight at -20°C. On the next day, RNA was recovered by centrifugation at 20,000 x g for 30 min at 4°C, washing with 80 % ethanol, air-drying the pellet and resuspending it at 66°C for 5 min in 30 µL NFW. The concentration of RNA was checked on a Nanodrop spectrophotometer and its presence and quality were checked on an 1.5 % agarose gel. If DNA contamination was observed the samples were treated a second time with DNAse.

Supplementary Tables

Supplementary Table 1: Primer sequences used in this study.

Primer name	Primer sequence	Binding site and product length(s)	Reference
upA_fwd	acgagccggaagcataaatgtaaagcaCCGAACGCCTCCAG TTGATG	Amplification of upstream (UP) fragment	This study
upA_rvs	taccagccctgccTTACGTTCCTACGTCAAGCAG		
downA_fwd	acgtaggaacgtaaGGCAGGGCTGGTATCAGCGAAA G	Amplification of downstream (DW) fragment	This study
downA_rvs	cacccgtggaaattaattaaggtaccgAATGCAGAGGCAGG CCCGAG		
8pEX_fwd	ACGGCAGGTAAGCTAATTCCAC	Binds to flanking region of inserts in pEXG2 plasmid	This study
8pEX_rvs	CCTCAACGACAGGAGCACGA		
9gen_fwd	TGCACATATGGCTCATCGCA	Binds to flanking regions of *saxA* gene in genome of 3D9	This study
9gen_rvs	AATGCGTCGTCGCTTCCT		
FWD_uidA	AACAGGTGGTTGCAACTGGA	Binds to β-d-glucuronidase gene in *E. coli*	[1]
REV_uidA	TTGCTGAGTTTCCCCGTTGA		[1]

Supplementary Table 2. Details of the analysis of 4MSOB-amine and 4MSOB-ITC by LC-MS/MS. Compounds were measured using an Agilent HPLC 1200/API3200 (AB SCIEX) instrument in positive ionisation mode. Abbreviations are: Q1, selected *m/z* of the first quadrupole; Q3, selected *m/z* of the third quadrupole; RT, retention time; DP, declustering potential (V); and CE, collision energy (V).

Q1	Q3	RT (min)	compound	DP	CE
136	72	0.5	4MSOB-amine	26	17
178	114	2.6	4MSOB-ITC	60	13

Supplementary Table 3: *saxA* and *saxB* expression in Ps. Log2FoldChanges (L2FC) and adjusted p-values (Wald test with Benjamin-Hochberg correction) of DESeq2 analyses of *saxA*, *saxB*, *saxC* genes.

Gene name / Locus Tag	**Condition**	**Effect**	**L2FC**	**padj**
***saxA* / MIFLLO_11060**	Psco	ITC	0.523789	0.00116082
	Psmono	ITC	0.629637	0.00569671
	PsnoITC	Partner	0.171169	0.35224
	PsITC	Partner	0.0511512	0.736944
***saxB* / MIFLLO_11060**	Psco	ITC	0.368971	0.0216534
	Psmono	ITC	0.441532	0.0451459
	PsnoITC	Partner	-0.0891716	0.600876
	PsITC	Partner	-0.196045	0.13862
***saxC* / MIFLLO_11070**	Psco	ITC	-0.0450266	0.641786
	Psmono	ITC	0.0502467	0.705504
	PsnoITC	Partner	0.805898	2.14999e-8
	PsITC	Partner	0.453799	0.000257485

Supplementary Table 4: *saxF* expression in Ps. Log2FoldChanges (L2FC) and adjusted p-values (Wald test with Benjamin-Hochberg correction) of DESeq2 analyses of *saxF*-associated efflux pump genes (Multidrug efflux pump subunit AcrB, with COG0841 annotation).

Locus Tag	Condition	Effect	L2FC	padj
MIFLLO_05915	Psco	ITC	0.0214242	0.915358
	Psmono	ITC	0.118351	0.370856
	PsnoITC	Partner	0.584841	3.50777e-8
	PsITC	Partner	0.468382	1.93891e-11
MIFLLO_11330	Psco	ITC	0.000448884	0.998677
	Psmono	ITC	0.0290503	0.832323
	PsnoITC	Partner	0.944021	4.28782e-10
	PsITC	Partner	0.832762	6.43417e-12
MIFLLO_13095	Psco	ITC	-0.00464959	0.984911
	Psmono	ITC	0.0523958	0.697142
	PsnoITC	Partner	-0.321806	0.00606728
	PsITC	Partner	-0.464192	1.33456e-7
MIFLLO_14945	Psco	ITC	-0.000697519	0.997347
	Psmono	ITC	0.0491537	0.641425
	PsnoITC	Partner	0.133161	0.431736
	PsITC	Partner	-0.0915416	0.577871
MIFLLO_15950	Psco	ITC	-0.0216916	0.885046
	Psmono	ITC	0.0252796	0.848564
	PsnoITC	Partner	0.133161	0.431736
	PsITC	Partner	0.120148	0.397794
MIFLLO_18075	Psco	ITC	0.0126581	0.928562
	Psmono	ITC	0.0310502	0.809603
	PsnoITC	Partner	-0.00689545	0.970914
	PsITC	Partner	-0.0620324	0.692812
MIFLLO_21925	Psco	ITC	-0.0351024	0.785644
	Psmono	ITC	-0.00701026	0.977402
	PsnoITC	Partner	0.0381757	0.790674
	PsITC	Partner	-0.0458315	0.694308
MIFLLO_25910	Psco	ITC	-0.0158039	0.927966
	Psmono	ITC	-0.0865286	0.46315
	PsnoITC	Partner	-1.47936	1.70152e-16
	PsITC	Partner	-1.26254	7.3265e-47

Supplementary Table 5. Reaction of Pl to 4MSOB-ITC. Log_2FoldChanges (L2FC) and adjusted p-values (Wald test with Benjamin-Hochberg correction) of DESeq2 analyses of Pl's response to 4MSOB-ITC in monoculture.

Locus Tag	L2FC	padj	protein annotation from whole genome
ACEBMG_12325	-2,725	6,71E-08	Aldehyde dehydrogenase B
ACEBMG_11515	-2,715	2,95E-16	hydroxymethylglutaryl-CoA reductase, degradative
ACEBMG_12320	-2,671	1,24E-51	UDP-glucose 4-epimerase
ACEBMG_11450	-2,288	5,30E-56	PepSY domain-containing protein
ACEBMG_09335	-2,271	1,74E-37	3-oxopropanoate dehydrogenase
ACEBMG_17620	2,003	1,35E-57	Alcohol dehydrogenase
ACEBMG_12995	2,053	8,79E-77	NADPH dehydrogenase
ACEBMG_15675	2,128	7,56E-71	N-ethylmaleimide reductase
ACEBMG_17225	2,129	4,12E-10	HxlR family transcriptional regulator
ACEBMG_16910	2,148	3,29E-58	HTH-type transcriptional regulator CmtR
ACEBMG_17655	2,191	1,51E-20	Alpha/beta fold hydrolase
ACEBMG_15290	2,194	1,96E-83	NADPH:quinone reductase
ACEBMG_03985	2,224	1,56E-89	Nitroreductase
ACEBMG_17650	2,255	1,40E-57	Pyrroline-5-carboxylate reductase catalytic N-terminal domain-containing protein
ACEBMG_17080	2,280	2,06E-23	Copper chaperone
ACEBMG_03025	2,666	1,29E-27	AraC family transcriptional regulator
ACEBMG_01540	2,672	4,88E-37	NAD-dependent epimerase/dehydratase family protein

Supplementary Figures

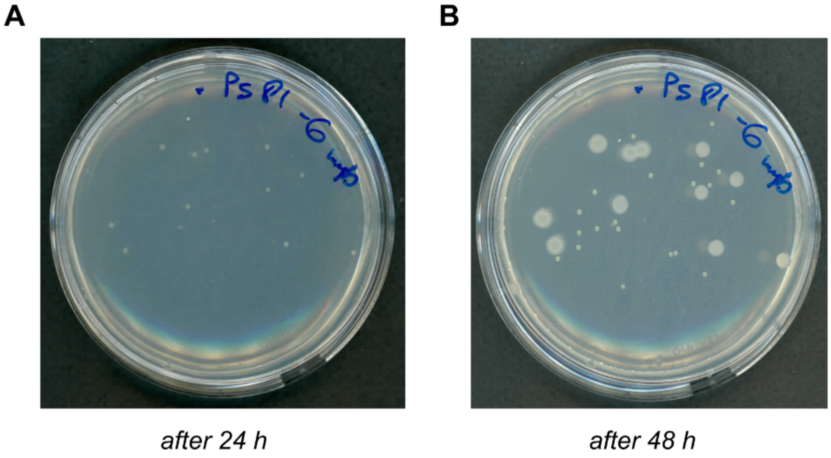

Supplementary Figure 1: Ps and Pl can be differentiated on R2A agar. Ps and Pl can be quantified separately based on morphology and the timepoint of appearance. Ps appears on R2A after 24 h of incubation at 30°C as whitish, irregular shaped colonies (**A**) and Pl appears only after 48 h as yellow, circular, convex colonies that are at this timepoint much smaller than Ps (**B**).

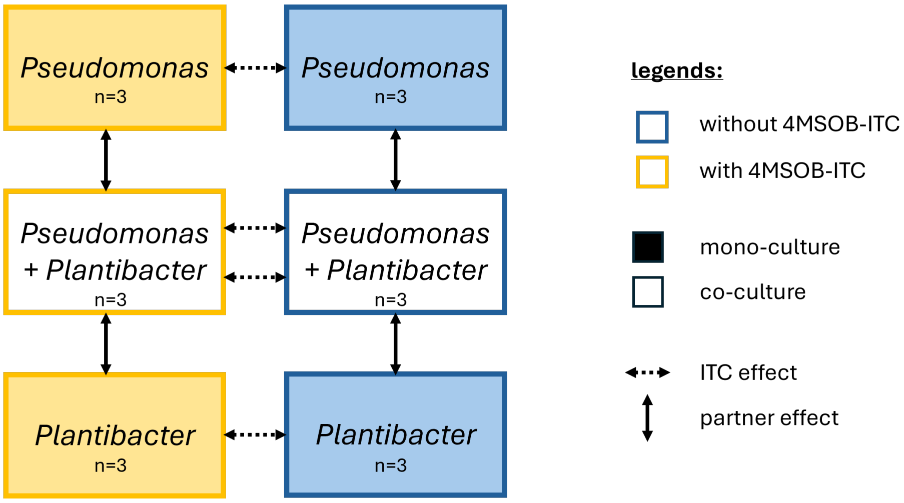

Supplementary Figure 2: Experimental design of Transcriptomics experiment. Ps and Pl were inoculated in R2A broth supplemented with 15 µg/mL 4MSOB-ITC either in mono- or co-culture (n=3 for each condition). Samples for RNA extraction were taken after 17-18 h. Arrows in the schematic drawing indicate possible comparisons for differential abundance analysis. Dotted arrows show comparisons depending on ITC exposure, solid arrows show comparisons depending on a partner strain. Yellow boxes illustrate R2A broth with ITC, blue boxes without ITC. Filled boxes depict mono-cultures, empty boxes co-cultures of Ps and Pl.

A

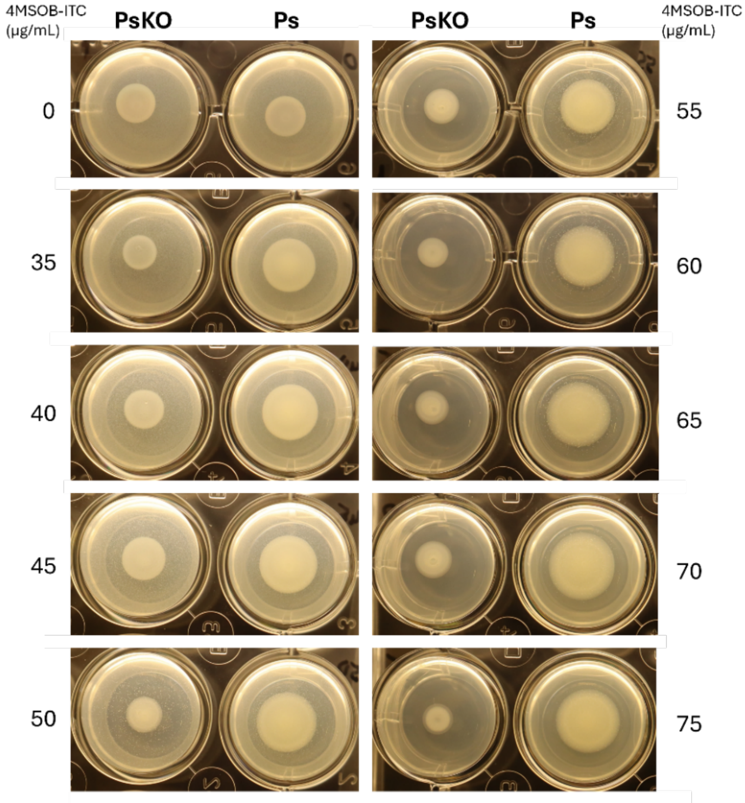

B

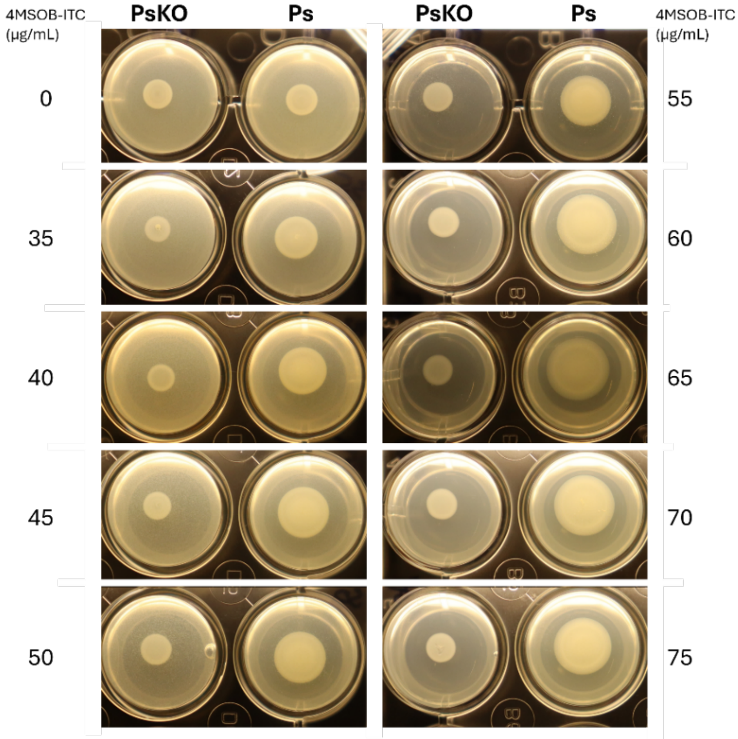

Supplementary Figure 3: Public good assay with Pl and Ps/PsKO. Pl was poured into R2A agar supplemented with different concentrations of 4MSOB-ITC (final OD = 0.001). 2 µL droplets of Ps or PsKO (OD=0.2) were drop spotted on top. Incubation for up to 4 days at 30°C. Pl appears cloudy in the agar at 0 µg/mL, individual colonies form with increasing ITC concentration and disappear at high concentrations (70-75 µg/mL). A close-up is depicted in main Fig. 1F.

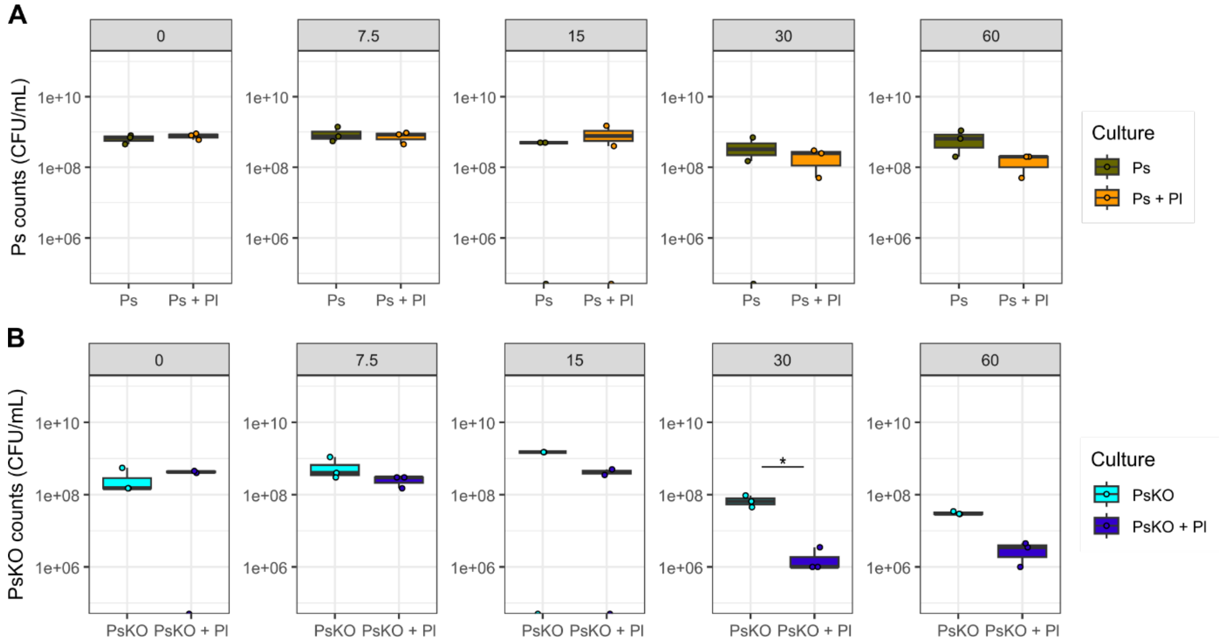

Supplementary Figure 4: Endpoint Ps and PsKO counts. Bacterial loads of Ps (A) and PsKO (B) after 22 h of incubation at 30°C in R2A broth supplemented with 0-60 µg/mL 4MSOB-ITC. Both strains were cultured alone or in co-culture with Pl (n=3). Significant comparisons at all timepoints are shown as asterisks * p<0.05, ** p<0.01, *** p<0.001 (two-sided t-test, fdr adjusted).

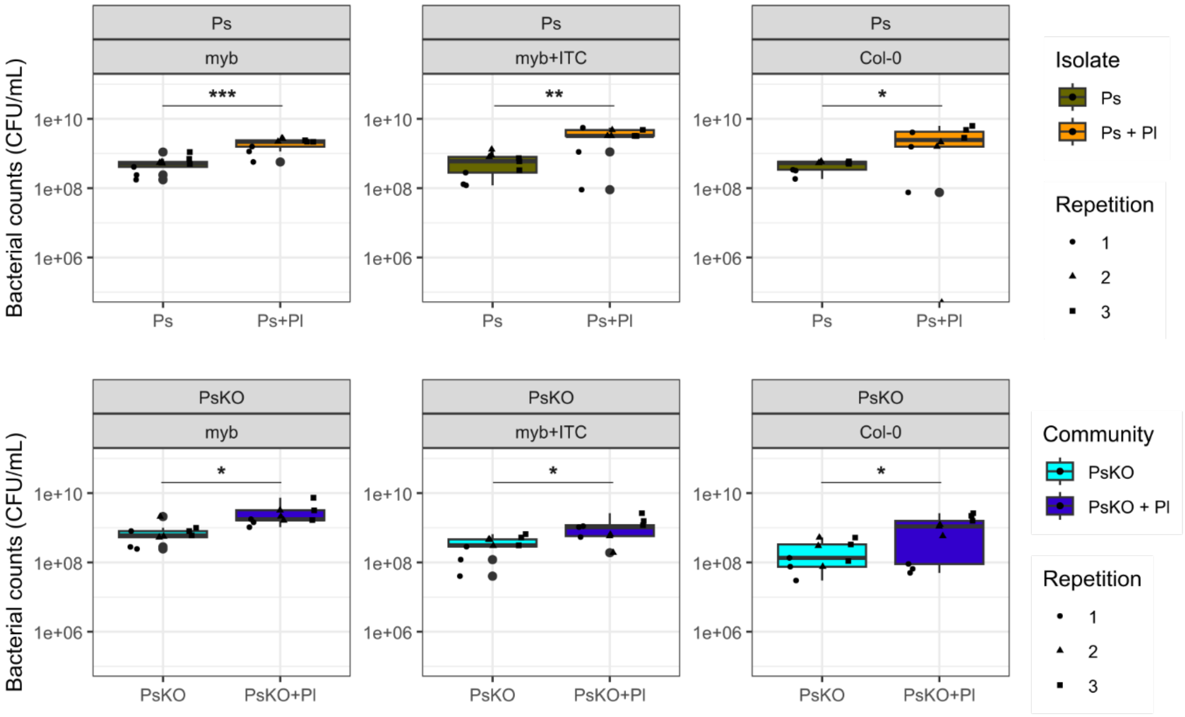

Supplementary Figure 5: Bacterial loads in leaf extract medium. Ps and PsKO counts (CFU/mL) in mono- or co-culture with Pl after incubation in leaf extract medium for 23-24 h at 30 °C, 220 rpm on the shaker. myb = *myb28/29* leaf extract medium without aliphatic GLS breakdown products. myb+ITC = *myb28/29* leaf extract medium supplemented with 60 µg/mL 4MSOB-ITC. Col-0 = Col-0 leaf extract medium. Pl counts of the same co-cultures are shown in Fig. 2B. Significant comparisons are shown as asterisks * p<0.05, ** p<0.01, *** p<0.001 (two-sided t-test, fdr adjusted).

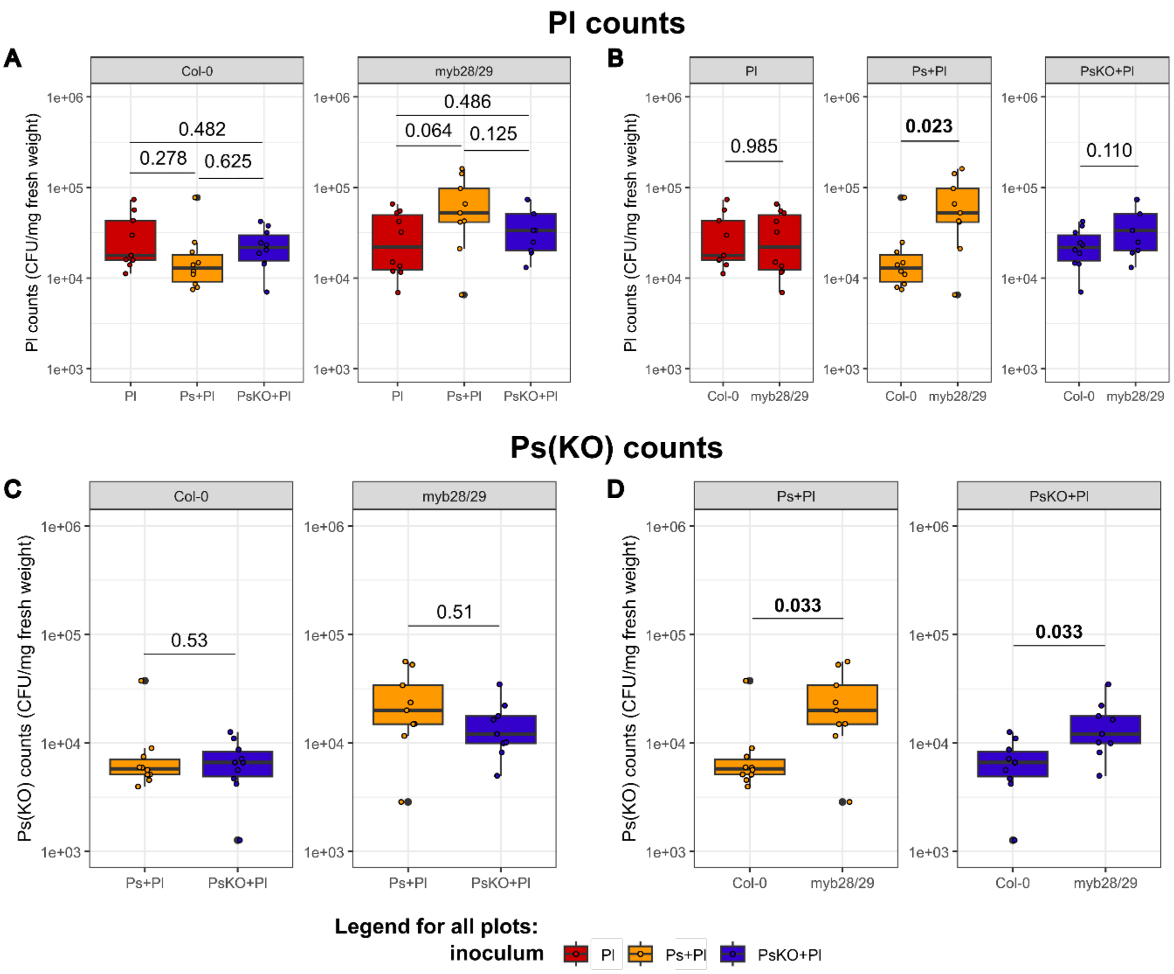

Supplementary Figure 6: Bacterial loads of Pl, Ps and PsKO in living leaf tissue. Three week-old gnotobiotic *A. thaliana* Col-0 plants or aliphatic GLS-free mutants *myb28/29* were mechanically wounded with scissors to release GLS breakdown products before inoculating them with OD_{600}=0.02 of Ps and Pl, PsKO and Pl or Pl in mono-culture. The rosettes were harvested 24h after inoculation and CFUs of Ps/PsKO (**C,D**) and Pl (**A,B**) were determined on R2A and R2A supplemented with 1.5 µg/mL kanamycin, respectively (n=9-10). Panels A and B, and C and D show the same data but faceted differently to either show the comparison between bacterial inocula (all not significant; **A,C**) or the comparisons between plant genotypes (**B,D**). p-values for two-sided t-test, fdr adjusted, are shown.

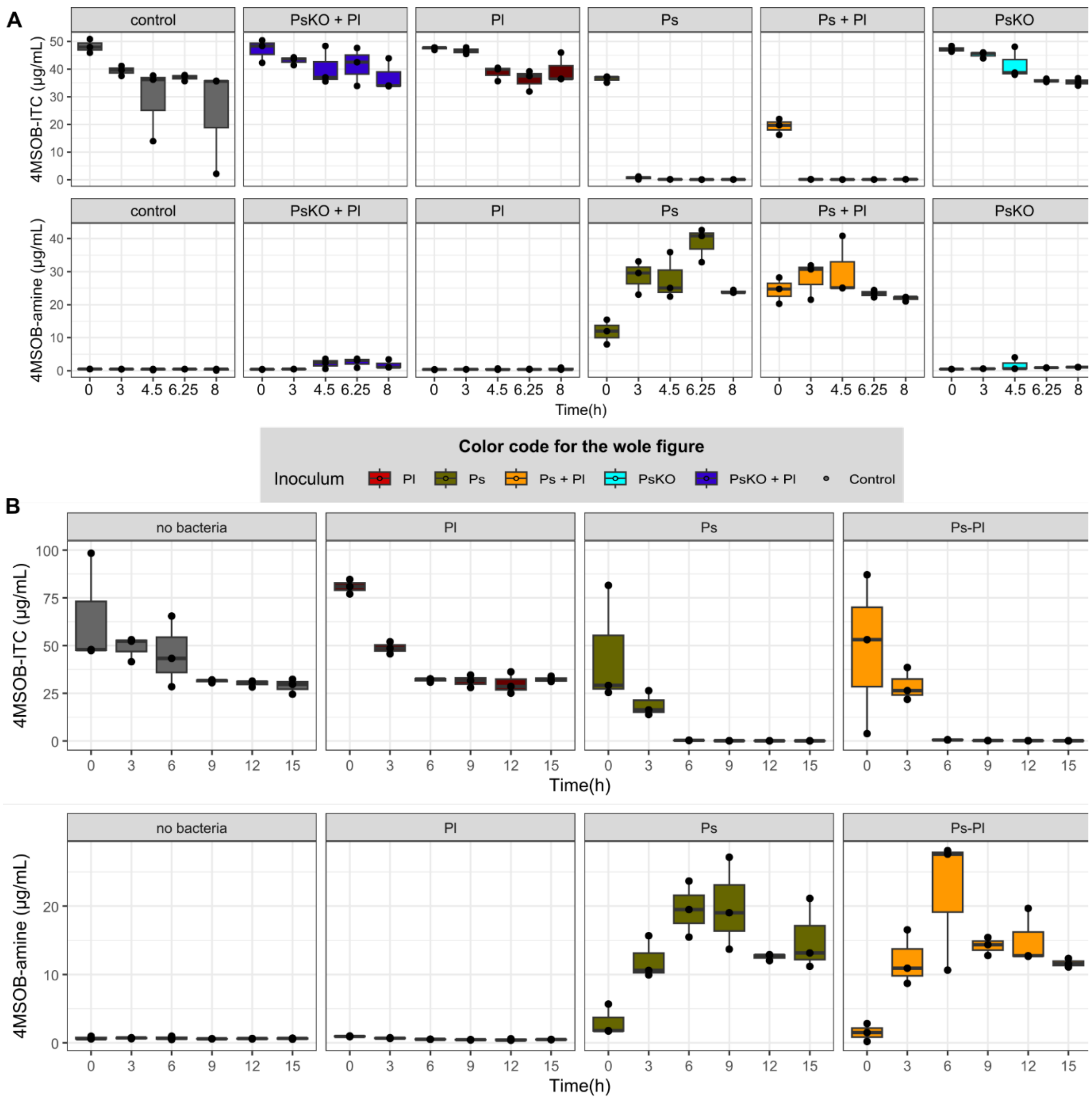

Supplementary Figure 7: 4MSOB-ITC and 4MSOB-amine quantification with HPLC-MS over time. 4MSOB-ITC and -amine were measured in bacterial supernatants over the course of 8 h (**A**) and 15 h (**B**). Starting concentrations were either 60 µg/mL (A) and 30 µg/mL (B) 4MSOB-ITC. Controls were not inoculated with bacteria but with sterile medium, all samples and controls n=3.

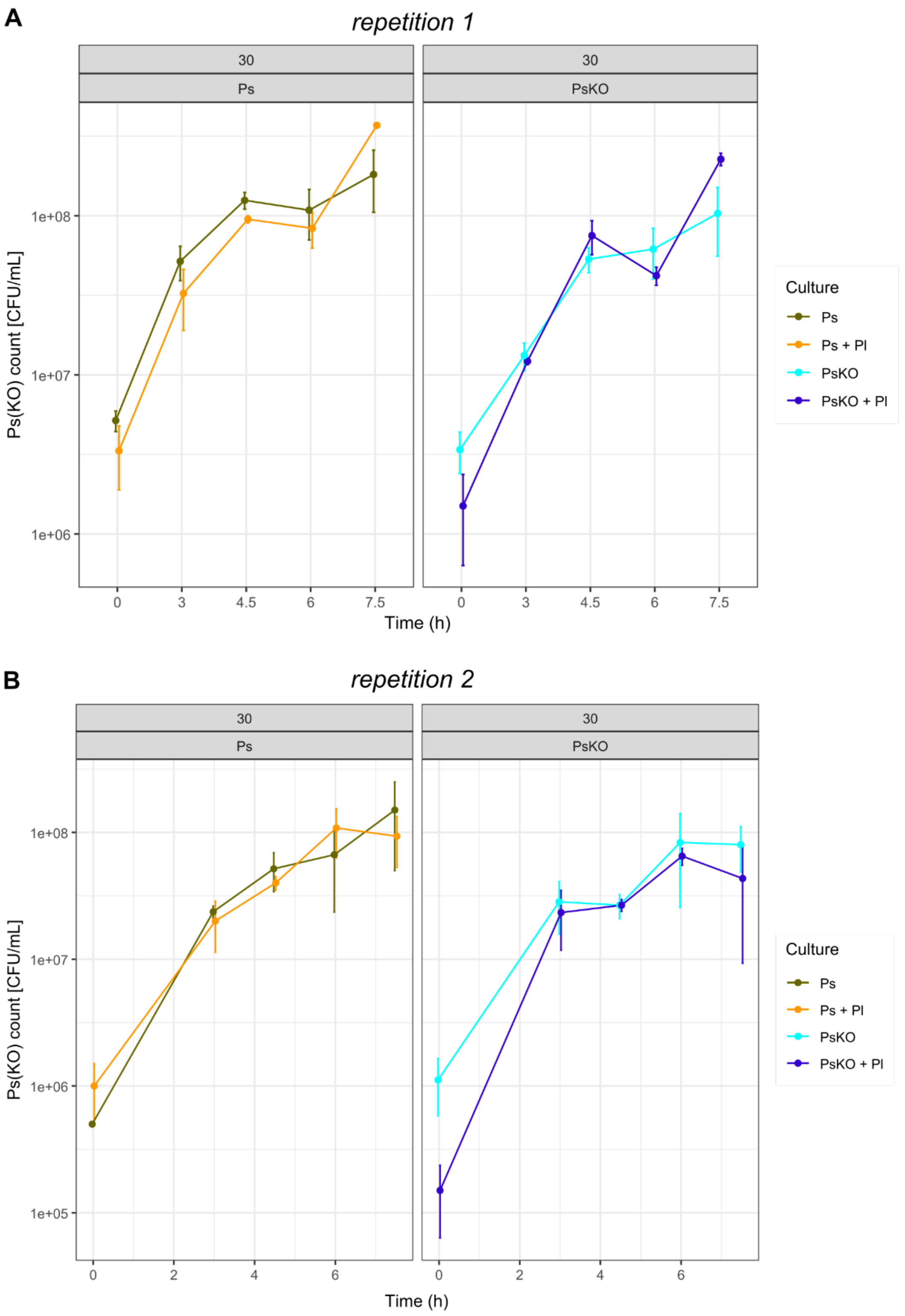

Supplementary Figure 8: Ps and PsKO counts with 30 µg/mL 4MSOB-ITC over time. Growth curves of mono- and co-cultures of Ps, and PsKO at 30 µg/mL 4MSOB-ITC in R2A broth at 30°C (mean ± standard deviation, n=3). The strains were inoculated with OD=0.2, the initial inoculum of the co-cultures was OD=0.4. The OD_{600} was measured over the course of 7.5 h. Controls were inoculated with sterile medium and did not show growth until the end of the experiment. A and B show two independent repetitions of the experiment. Pl counts of the same experiments are shown in Fig. 3B.

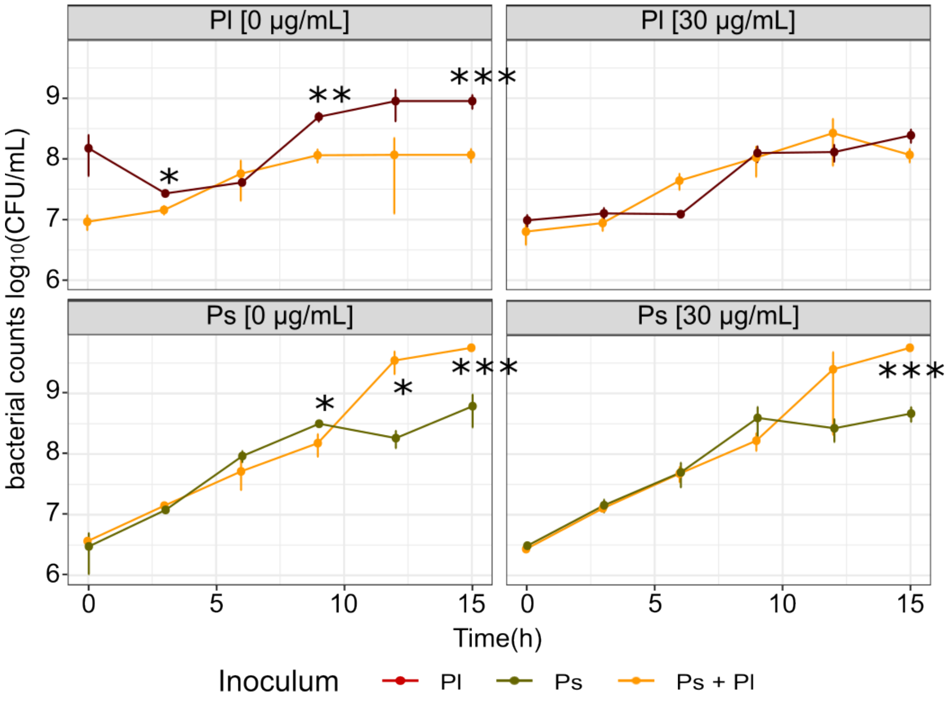

Supplementary Figure 9: Bacterial counts of Ps and Pl mono- and co-culture over the course of 15h. Strains were grown in R2A broth supplemented with either 0 or 30 µg/mL 4MSOB-ITC in mono- or co-cultures and samples over time (mean ± standard deviation, n=3 technical replicates, one repetition). Colours visualize the inoculated culture, facets indicated the counted strain at different ITC concentrations. Significant comparisons at all timepoints are shown as asterisks * $p<0.05$, ** $p<0.01$, *** $p<0.001$ (t-test, fdr adjusted).

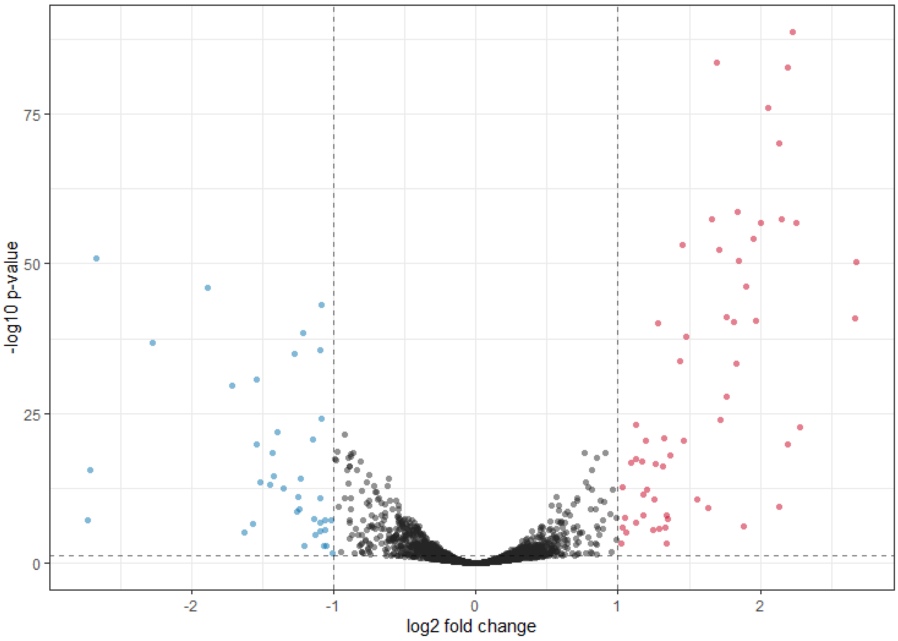

Supplementary Figure 10: Pl's reaction to 4MSOB-ITC in monoculture. Volcano plot of significant DEGs (adjusted p-value < 0.05, |L2FC| >1). Significant genes are shown in blue (downregulated) or red (upregulated).

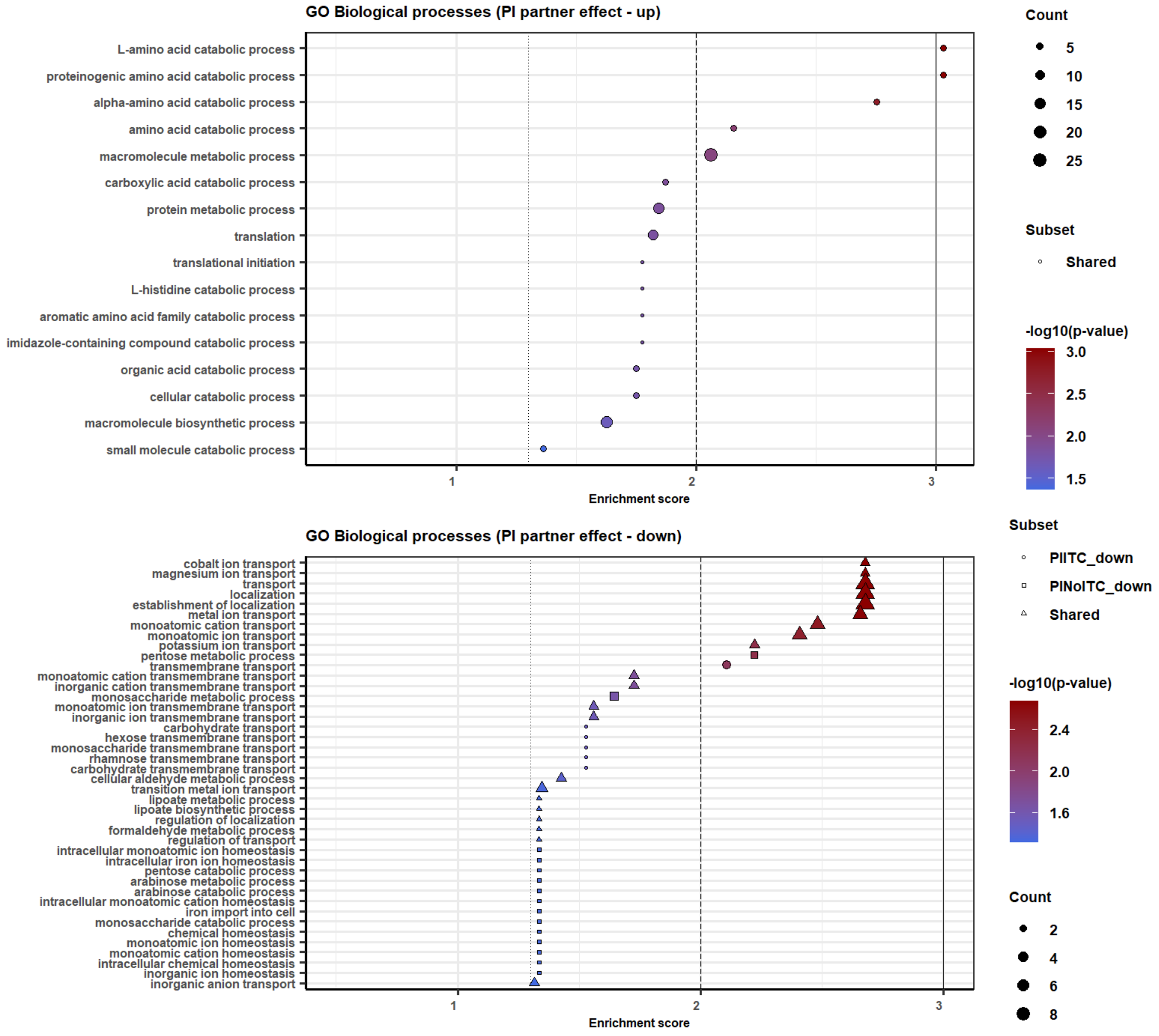

Supplementary Figure 11: Ps's reaction to Pl dependent on 4MSOB-ITC exposure. (A) Enriched GO terms for up-regulated Ps DEGs. (B) Enriched GO terms for down-regulated DEGs. The shape shows whether a term was enriched only with or without 4MSOB-ITC or independent of it. The color indicates the p-value of the Fisher test, horizontal lines depict p=0.001, p=0.01 and p=0.05. The size shows how many GO terms were identified for each biological process.

Supplementary References

1. Schlechter RO, Jun H, Bernach M *et al.* Chromatic bacteria – A broad host-range plasmid and chromosomal insertion toolbox for fluorescent protein expression in bacteria. *Front Microbiol* 2018;**9**:423623.

2. Kremer JM, Sohrabi R, Paasch BC *et al.* Peat-based gnotobiotic plant growth systems for Arabidopsis microbiome research. *Nat Protoc* 2021;**16**:2450–70.

3. Hoagland DR, Arnon DI. The water-culture method for growing plants without soil. *California Agricultural Experiment Station*:347.

4. Textor S, Gershenzon J. Herbivore induction of the glucosinolate–myrosinase defense system: major trends, biochemical bases and ecological significance. *Phytochem Rev* 2009;**8**:149–70.
